# JAKs and STATs from a Clinical Perspective: Loss-of-Function Mutations, Gain-of-Function Mutations, and Their Multidimensional Consequences

**DOI:** 10.1007/s10875-023-01483-x

**Published:** 2023-05-04

**Authors:** Nils Ott, Laura Faletti, Maximilian Heeg, Virginia Andreani, Bodo Grimbacher

**Affiliations:** 1 Institute for Immunodeficiency, Center for Chronic Immunodeficiency (CCI), Medical Center, Faculty of Medicine, University of Freiburg, Freiburg, Germany; 2 Division of Biological Sciences, Department of Molecular Biology, University of California, La Jolla, San Diego, CA USA; 3 Clinic of Rheumatology and Clinical Immunology, Center for Chronic Immunodeficiency (CCI), Medical Center, Faculty of Medicine, University of Freiburg, Freiburg, Germany; 4 DZIF – German Center for Infection Research, Satellite Center Freiburg, Freiburg, Germany; 5 CIBSS – Centre for Integrative Biological Signalling Studies, University of Freiburg, Freiburg, Germany; 6 RESIST – Cluster of Excellence 2155 to Hanover Medical School, Satellite Center Freiburg, Freiburg, Germany

**Keywords:** JAK/STAT signaling pathway, Loss-of-function mutations, Gain-of-function mutations, STAT1, STAT3, STAT6, JAK1, JAK3, Clinical phenotype, Immunodeficiency, Autoimmunity, Autoinflammation, Inborn errors of immunity

## Abstract

The JAK/STAT signaling pathway plays a key role in cytokine signaling and is involved in development, immunity, and tumorigenesis for nearly any cell. At first glance, the JAK/STAT signaling pathway appears to be straightforward. However, on closer examination, the factors influencing the JAK/STAT signaling activity, such as cytokine diversity, receptor profile, overlapping JAK and STAT specificity among non-redundant functions of the JAK/STAT complexes, positive regulators (e.g., cooperating transcription factors), and negative regulators (e.g., SOCS, PIAS, PTP), demonstrate the complexity of the pathway’s architecture, which can be quickly disturbed by mutations. The JAK/STAT signaling pathway has been, and still is, subject of basic research and offers an enormous potential for the development of new methods of personalized medicine and thus the translation of basic molecular research into clinical practice beyond the use of JAK inhibitors. Gain-of-function and loss-of-function mutations in the three immunologically particularly relevant signal transducers STAT1, STAT3, and STAT6 as well as JAK1 and JAK3 present themselves through individual phenotypic clinical pictures. The established, traditional paradigm of loss-of-function mutations leading to immunodeficiency and gain-of-function mutation leading to autoimmunity breaks down and a more differentiated picture of disease patterns evolve. This review is intended to provide an overview of these specific syndromes from a clinical perspective and to summarize current findings on pathomechanism, symptoms, immunological features, and therapeutic options of STAT1, STAT3, STAT6, JAK1, and JAK3 loss-of-function and gain-of-function diseases.

## Mechanisms of the JAK/STAT Signaling Pathway

The JAK/STAT signaling pathway is a prime example of the transmission of signals from extracellular ligands to the nucleus. It can explain how messenger substances such as cytokines and growth factors can mediate their functions into the cell in a diverse but specific manner. To date, more than 50 cytokines, growth factors, and hormones are known to trigger cell responses, using the signaling module of Janus tyrosine kinase (JAK) and signal transducer and activators of transcription (STAT) molecules [1].

**Fig. 1:**
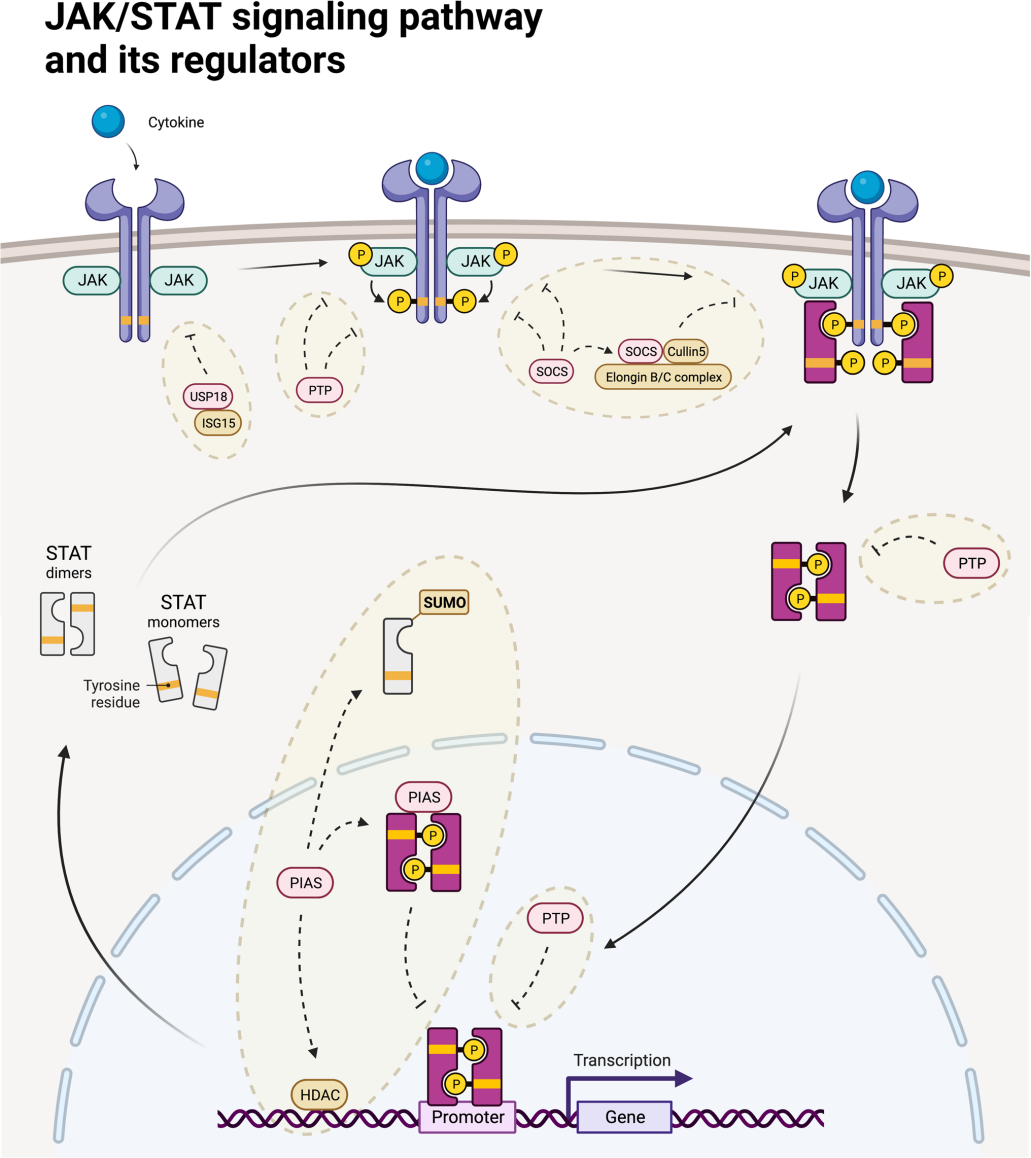
The traditional Janus kinase (JAK)/signal transducer and activator of transcription (STAT) pathway and its regulators. The activation of JAKs after cytokine stimulation results in the phosphorylation of STAT monomers or dimers, which then become activated, and translocate as dimers to the nucleus to activate gene transcription. Several proteins are involved in the regulation of the pathway: protein inhibitors of activated STATs (PIAS) can bind to STAT dimers and inhibit their DNA binding capacity, induce SUMOylation of STATs, or enhance the recruitment of other STAT co-repressors such as histone deacetylases (HDAC). Protein tyrosine phosphatases (PTPs) induce dephosphorylation of STATs and JAKs at various locations in the cell. Suppressors of cytokine signaling (SOCS) bind via their SH2 domain to phosphorylated tyrosine residues of JAKs, thereby inhibiting their kinase activity and the recruitment of STATs. In addition, they can activate other molecules such as Elongin B/C or Cul5, initiating an ubiquitylation cascade resulting in proteasomal degradation. ISG15 stabilizes USP18 which can disrupt the interaction of JAK and a cytokine receptor (IFNAR2). Created with BioRender.com

In general, the binding of an extracellular ligand to a cytokine receptor results in the activation of receptor-associated JAKs (Fig. 1). These tyrosine kinases then phosphorylate themselves (autophosphorylation) and their associated receptors (transphosphorylation) to allow the recruitment of inactive STAT monomers via the SH_2_ domains of STATs. STATs are normally present as inactive monomers or unphosphorylated dimers in the cytoplasm before being recruited into the JAK/receptor complex [2]. Immediately after STAT monomers have bound to the JAK/receptor complex, they themselves become phosphorylated (pSTAT) and dimerize due to spatial proximity. STAT dimers can then translocate into the nucleus where they bind to specific DNA sequences in the promoters of STAT pathway target genes to activate gene transcription (Fig. 1) [3].

The traditional concept of JAK/STAT signaling has been overturned several times by findings of, e.g., most STAT molecules residing as inactive cytoplasmatic STAT dimers, STAT monomers binding to DNA, or unphosphorylated STATs promoting gene transcription [2, 4-7]. The observations of non-canonical effects complement the canonical JAK/STAT signaling pathway but add even more complexity to the already highly regulated machinery involving regulatory proteins such as protein inhibitors of activated STATs (PIAS), protein tyrosine phosphatases (PTPs), and suppressors of cytokine signaling (SOCS) (Fig. 1). These negative regulators control amplitude and kinetics of STAT activity via various mechanisms, thereby ensuring balanced signaling in cells. PIAS proteins can bind STAT dimers specifically blocking the DNA binding capability of the dimers [8]. In addition, PIAS are involved in SUMOylation and the recruitment of other STAT co-repressors such as histone deacetylases (HDAC) [9, 10]. PTPs are essential restorers of the signaling cascade by sequestering JAKs or STATs (e.g., SHP1 and SHP2 by their SH_2_ domain), inducing their dephosphorylation, thus counteracting prolonged activation and sensitizing the molecules to a new phosphorylation stimulus instead. Of central importance are the STAT-induced STAT inhibitors called SOCS, which, on the one hand, bind to phosphorylated tyrosine residues of JAKs via their SH_2_ domain and thereby prevent both the recruitment of STATs and the kinase activity of JAKs [11, 12]. On the other hand, SOCS proteins mark STATs for proteasomal degradation by the SOCS box motif [13, 14], which can recruit factors including Elongin B/C, Cul5, RBX1, and RBX2 initiating the ubiquitylation cascade [15-17]. Other factors involved in, e.g., type I interferon (IFN) signaling regulation are ubiquitin-specific peptidase 18 (USP18) and interferon-stimulated gene 15 (ISG15), whose expression is itself type I/III IFN induced. USP18 acts as a negative regulator of the type I IFN signaling pathway via interferon-α/β receptor (IFNAR), JAK1 and TYK2, and STAT1 and STAT2, primarily preventing autoinflammation due to overshoot of the type I IFN response. USP18 can bind to the receptor subunit IFNAR2, thereby removing JAK1 from the receptor [18]. The receptor complex is disrupted which leaves the cells with reduced type I interferon sensitivity resulting in a refractory state. Through its enzymatic function as an isopeptidase, USP18 is involved in the deISGylation of ISG15. While ISG15 can act as an extracellular cytokine promoting the production of type II IFN in natural killer cells, it has multiple cellular functions that contribute to viral defense in particular, i.a., inhibition of viral protein function and interaction with host proteins [19]. Notably, stabilization of USP18 by free ISG15 protects against its proteasomal degradation; thus, ISG15 supports the downregulatory function of USP18 in the control of type I IFN-JAK/STAT signaling pathway [20].

**Fig. 2:**
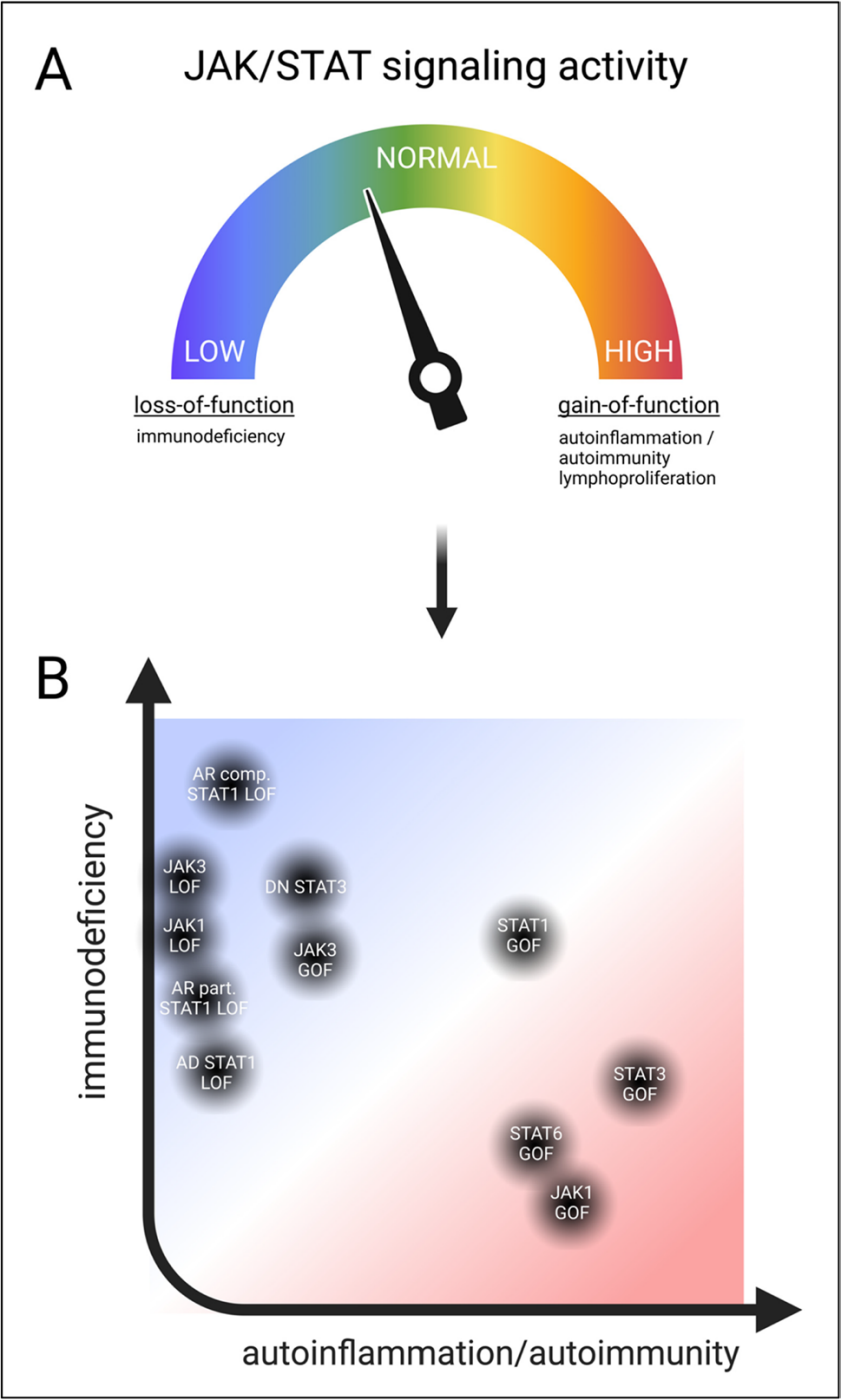
JAK/STAT signaling activity represented in different ways of understanding. **A** Traditionally, JAK/STAT signaling activity was understood as a spectrum. Herein, immune cells maintain the balance between the two possible extremes, immunodeficiency and autoinflammation/autoimmunity at a normal level. Loss-of-function or gain-of-function mutations of the various players can alter the balance, resulting in insufficient or exceeding JAK/STAT activity. **B** A deeper understanding of the pathomechanisms allows a more differentiated view of the diseases today. Immunodeficiency and autoimmunity symptoms are not mutually exclusive but may have shared molecular causes. JAK/STAT LOF and GOF diseases can therefore be represented two-dimensionally in a coordinate system with different intensities of the two symptom complexes. LOF, loss-of-function; GOF, gain-of-function; DN, dominant-negative. Created with BioRender.com

**Fig. 3:**
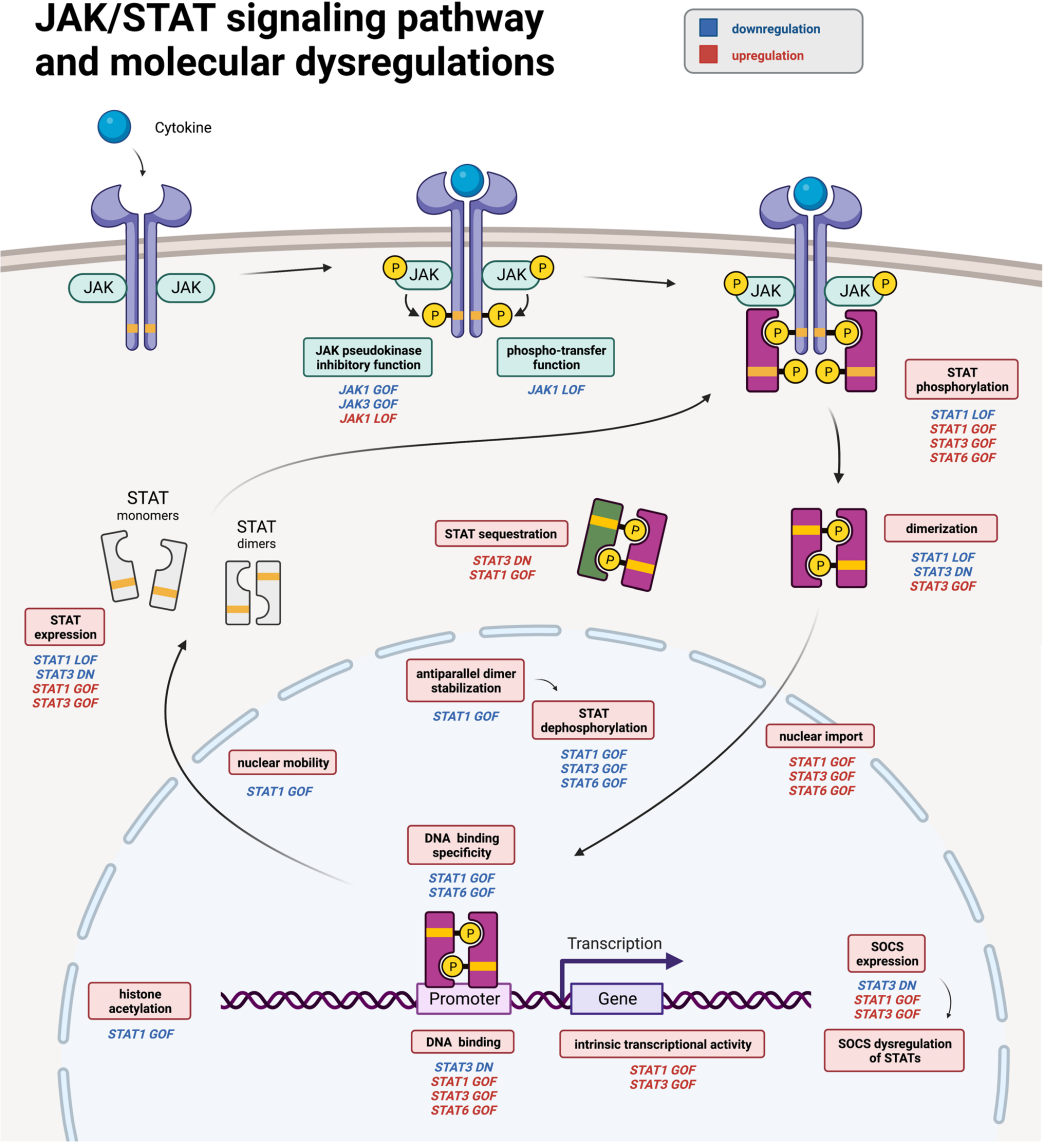
The traditional Janus kinase (JAK)–signal transducer and activator of transcription (STAT) pathway and molecular dysregulations. At a molecular level, a range of possible dysregulations can occur in JAK/STAT LOF and GOF diseases. Illustrated here are most of the molecular pathomechanisms that have been investigated to date for the respective syndromes. Defects of the pseudokinase domain of JAKs can occur, some of which may result in disruption of the phosphor-transfer function of JAK. Also, defects of STAT phosphorylation, dimerization, or binding of other STATs until their sequestration affect STAT activity. Furthermore, STAT dimers are known to get destabilized in their antiparallel conformation which can promote STAT dephosphorylation. Altered nuclear import and mobility can cause nuclear accumulation of active STAT dimers. Variations in DNA binding specificity, DNA binding affinity, epigenetic changes, and transcriptional activity eventually lead to increased, decreased, or non-specific expression of downstream target genes. Resulting altered SOCS expression with dysregulations of STATs is just one mechanism how JAK/STAT LOF and GOF can have a further effect. STAT isoform-specific dysregulations at the cellular level or their influence on further downstream pathways will be discussed in the corresponding sections. Downregulations are indicated in blue; upregulations are indicated in red. Created with BioRender.com

**Fig. 4:**
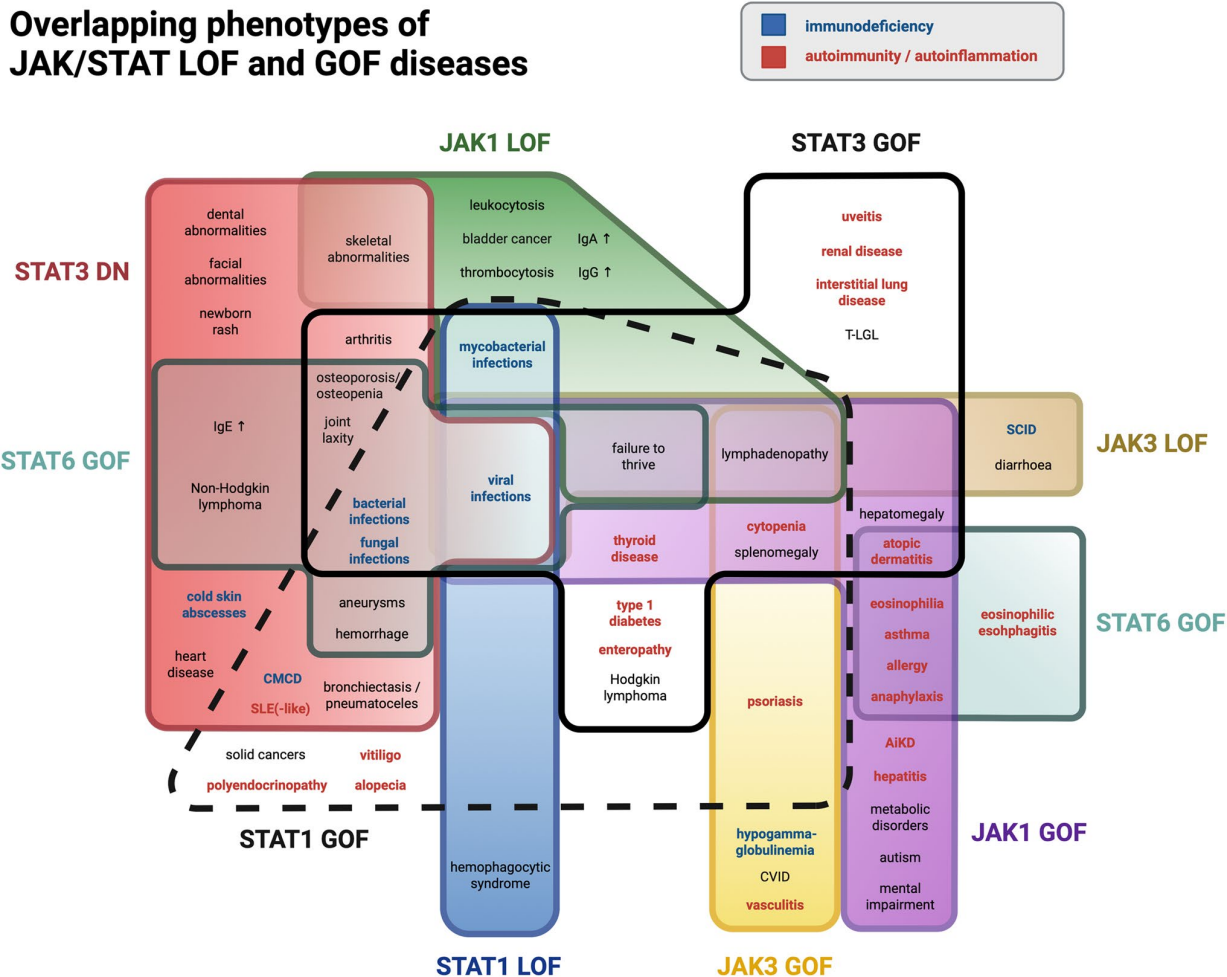
Overlapping phenotypes of JAK/STAT LOF and GOF diseases. Visual representation of the clinical phenotypes in JAK/STAT LOF and GOF diseases. Each disease can manifest with typical symptoms, which can affect different organ systems. Some syndromes show more overlapping symptoms than others. Symptoms can also occur in both LOF and GOF of the same protein, but also in LOF and GOF of different proteins. Rather immunodeficiency associated symptoms appear blue; rather autoimmunity associated symptoms appear red. STAT6 GOF symptoms are displayed in two boxes. LOF, loss-of-function; GOF, gain-of-function; IgE, Immunoglobulin E; IgA, immunoglobulin A; IgG, Immunoglobulin G; CMCD, chronic mucocutaneous candidiasis disease; SLE, systemic lupus erythematosus; CVID, common variable immunodeficiency; T-LGL, T cell large granular lymphocyte leukemia; SCID, severe combined immunodeficiency; AiKD, autoinflammation keratinization disease. Created with BioRender.com

Immune homeostasis in humans is important to avoid the two extremes of immunodeficiency and autoimmunity/autoinflammation. Signal transducers and activators of transcription molecules are “immunorheostats” that prevent such diseases by regulating the innate and adaptive immune system (Fig. 1) [21]. As can be seen, the regulatory mechanisms of STAT balance are complex and crucially dependent on, i.a., JAKs. A few years ago, the possible pathological mutations were classified into a spectrum ranging from insufficient to excessive immune responses (Fig. 2A). In the following section, nine of these disease-causing mutations shall be discussed as examples, including their molecular dysregulations (Fig. 3) as well as clinical manifestations (Fig. 4, Table 1). Furthermore, we will emphasize the idea that our understanding of these diseases is evolving from a continuum of immunodeficiency vs. autoimmunity/autoinflammation to a two-dimensional concept (Fig. 2B). We move away from the one-dimensional Either/Or and instead reimagine advanced representations to account for the complexity of JAK/STAT pathway dysregulations.

**Table 1:** Overview of phenotypes associated with JAK/STAT mutations

Molecule	Mutation	Predominant phenotype
STAT1	LOF	*Partial AR*: mild mycobacterial and viral infections *Complete AR*: fatal mycobacterial and viral infections *AD*: mycobacterial infections
	GOF	Chronic mucocutaneous candidiasis (CMCD) Increased susceptibility to bacterial or viral infections Autoimmunity IPEX-like phenotype
STAT2	LOF	Viral infections
	GOF	Autoinflammation (lethal)
STAT3	LOF	AD-hyper-IgE syndrome
	GOF	Multisystem autoimmunity, infections, growth failure *Somatic*: Large granular lymphocyte leukemia
STAT4	Polymorphisms	Rheumatoid arthritis Systemic lupus erythematosus
STAT5a/5b	LOF	*AR*: failure to thrive, immunodeficiencies (e.g., generalized eczema, thrombocytopenic purpura, and respiratory infections) Autoimmunity
	GOF	Large granular lymphocyte leukemia
STAT6	LOF	Not reported
	GOF	Atopic dermatitis, allergy, elevated serum IgE
JAK1	LOF	Recurrent mycobacterial infections, early-onset bladder cancer
	GOF	Multiorgan immunodysregulation, AML, ALL, other malignancies
JAK2	LOF	Not reported
	GOF	PCV, PMF, ET, CML
JAK3	LOF	*AR*: T^−^B^+^NK^–^SCID
	GOF	CTLA4-dependent immunodysregulatory syndrome, NK cell lymphoproliferation syndrome, AML, ALL, lymphoma
TYK2	LOF	Increased susceptibility to intracellular bacteria (especially mycobacteria), repeated viral infection, eczema
	GOF	Malignancies

Certain loss-of-function and gain-of-function mutations lead to typical phenotypes. The resulting disorders recorded are listed. Some mutations have not yet been described in humans. *LOF*, loss-of-function; *GOF*, gain-of-function; *AR*, autosomal recessive; *AD*, autosomal dominant; *CMCD*, chronic mucocutaneous candidiasis disease; *CTLA4*, cytotoxic T lymphocyte-associated protein; *IPEX*, immunodysregulation polyendocrinopathy enteropathy X-linked; *AML*, acute myeloid leukemia; *ALL*, acute lymphoblastic leukemia; *PCV*, polycythemia vera; *PMF*, primary myelofibrosis; *ET*, essential thrombocythemia; *CML*, chronic myelogenous leukemia; SCID, severe combined immunodeficiency

## Heterozygous *STAT3* Dominant-Negative Mutations

### Overview

Among all seven STAT molecules, STAT3 is a key transcription factor for the immune system. For example, STAT3 is crucial for the differentiation of naïve CD4^+^ T cells into T_H_17 helper cells [22]. T_H_17 cells contribute significantly to the recruitment of effector cells to sites of inflammation caused by bacterial and fungal pathogens. By activating neutrophil granulocytes, this cell group enables effective control of bacteria and fungi in healthy individuals [23]. Heterozygous germline mutations may occur in *STAT3*, impairing the function of the gene product in different biochemical ways. The resulting molecules cause loss-of-function of the JAK/STAT3 pathway almost always by exerting a dominant-negative (DN) effect on functional STAT3 monomers sequestering them into defect STAT^WT^:STAT^mut^ dimers.

STAT3 mediates cytokine signaling responses of, for example, IL-6, IL-9, IL-10, IL-11, IL-17, IL-19, IL-20, IL-21, IL-22, IL-23, leukemia inhibitory factor (LIF), oncostatin M (OSM), interferon-α (IFN-α), IFN-β, or IFN-γ in development and the immune system. In many ways, the transcription factor thereby maintains an immunological balance between inflammation and immunodeficiency.

### Genotype

Most of the *STAT3* DN mutations are inherited or de novo missense mutations, but in-frame deletions, in-frame insertions, essential splice-site mutations, or even frameshift mutations have been documented. Most of the autosomal dominant DN mutations lay in the DNA binding domain or SH_2_ domain and impair STAT3 dimerization or the homodimers’ ability to bind to DNA in order to activate gene transcription [24-27]. As STAT3 forms homodimers, mutant STAT3 molecules can exert a DN effect on the wild-type STAT3 molecules and hence weaken the cytokine signaling [28]. Homozygous mutations in *STAT3* lead to embryonic lethality in *STAT3*^−/−^ knock-out mice, but this have not been reported in humans, whereas heterozygous *STAT3*^+/-^ mice appear phenotypically normal, at least when kept under specific-pathogen-free conditions. As STAT3 not only has key functions in the immune system but is also essential for development and cell survival, human biallelic LOF mutations are not compatible with life [29, 30]. Unlike in mice, there was the observation that in humans STAT3 haploinsufficiency could produce a phenotype: Natarajan et al. described STAT3 haploinsufficiency in a patient with a de novo splice-site mutation and a more atypical phenotype with fatal invasive aspergillosis, in which the *STAT3* variant S381* led to a reduction of STAT3 activity by 50% due to nonsense-mediated decay [31]. However, a large-scale functional analysis of all reported pathogenic *STAT3* variants revealed that > 95.3% of all variants exert a DN effect through aberrant proteins (caused by premature stop of translation, reinitiation of translation, or alternative transcripts) rather than a haploinsufficient effect [32]. Full penetrance of *STAT3* DN mutations is postulated, although intrafamilial phenotypic variation and at least three cases of unaffected patients are reported [33-35]. Possible explanations for the lack of a full clinical phenotype and intrafamilial phenotypic variation may be the young age of the patients at first examination and somatic mosaicism, respectively [34, 36]. Not all cases of HIES are caused by DN mutations in *STAT3*. Instead, defects in other molecules transducing or modulating STAT3 signaling may also cause a phenotype that is reminiscent of HIES. During the last years, additional monogenetic defects have been described, such as an autosomal recessive LOF mutation in different subunits of the IL-6 receptor (*IL6ST*, *IL6R*), impairing STAT3 function [37-39]. Patients with homozygous nonsense mutations in the zinc finger transcription factor *341* (*ZNF341*) gene show significantly reduced *STAT3* mRNA levels due to an impaired ability to bind to the *STAT3* promoter. ZNF341 is a key transcription factor regulating STAT3 expression. The subsequently impaired positive regulation of transcription of *STAT3* mimics a STAT3 deficiency. ZNF341 LOF patients present with similar clinical manifestations to those described in patients with *STAT3* DN mutations. Therefore, biallelic *ZNF341* LOF mutations are considered as an autosomal recessive cause of hyper-IgE syndrome (HIES) [40, 41]. Autosomal recessive mutations, in *DOCK8*, *PMG3*, or *SPINK5*, may also cause an HIES-like phenotype, however, with additional phenotypic complications [42]. STAT6 GOF also shows a remarkably overlapping phenotype with HIES, initiating a discussion on considering STAT6 GOF as another cause of AD-HIES (see below).

### Clinical Phenotype

The attenuated transduction of extracellular cytokine signals by STAT3 leads to a stereotypical phenotype called Job’s syndrome or HIES. The syndrome’s clinical manifestations have been first described in 1966 [43] and since then further characteristic symptoms have been recognized [44]. The primary immunodeficiency (PID) HIES comprises different features with a characteristic clinical triad: extremely elevated IgE levels (> 2000 U/ml), cyst-forming pneumonia, and typical recurrent staphylococcal skin abscesses with a lack of inflammation, therefore referred to as “cold abscesses” [43]. Sixty to 80% of patients present with a newborn rash in the first weeks of life, which may develop into eczematoid dermatitis and may persist into adolescence, making the newborn rash a typical early HIES symptom [27, 45-47]. Infections of the respiratory tract, especially pulmonary infections, play a major role for patients as these often show unsatisfactory healing. If prophylaxis or aggressive treatment of the pathogens (*Staphylococcus aureus*, *Streptococcus pneumoniae*, *Haemophilus influenzae*, non-tuberculous mycobacteria, gram-negative bacteria such as *Pseudomonas aeruginosa*) are not effective, chronic infections with bronchiectasis or pneumatoceles may develop, which increase morbidity and mortality and should therefore be prevented. The use of antibiotic and antifungal drugs is therefore indicated for prophylaxis but also for therapy. Besides the immunological abnormalities, extra-hematopoietic symptoms also exist. HIES patients present with a characteristic facial morphology including prominent forehead and chin, broad nose, and wide-set eyes. The majority of patients have a high-arched palate, retained primary teeth, and hyperextensible joints, as well as scoliosis, arthritis, and osteopenia or osteoporosis, resulting in bone fractures after minor trauma. The vascular system is also affected: Compared to healthy individuals, there is a higher incidence of aneurysms, vascular tortuosity, dilatation, and thickened walls of the coronary or cerebral vessels, which increases the risk of myocardial infarction, subarachnoid hemorrhage, or other bleedings. Patients should therefore undergo routine MRI screening on a regular basis [48]. The risk for malignancies, especially non-Hodgkin’s lymphoma, is also increased [49-51]; hence, screening for lymphadenopathies is recommended. A certain group of patients presents with autoimmune symptoms (see below).

### Molecular and Cellular Phenotype

Patients show eosinophilia (> 700/μl), low numbers of memory T cells and memory B cells, and almost no T_H_17 cells [52]. As already described, the STAT3-dependent development of these cells from CD4-naïve T cells is severely impaired by *STAT3* DN mutations. STAT3-mediated transcription of genes encoding IL-17 and the transcription factor RAR-related orphan receptor gamma-t (RORγt) is essential since they define T_H_17 differentiation [53-57]. Their presence in the tissue is, however, necessary to maintain epithelial immunity against bacterial and fungal pathogens. Chronic mucocutaneous candidiasis (CMC) affecting nails, oropharynx, esophagus, and the vagina is therefore a common complication [44, 58]. Opportunistic pathogens such as *Pneumocystis jirovecii* [59] and disseminated infections of the gastrointestinal tract (*Histoplasmosis*, *Cryptococcus*) or meningitis (*Coccidiodes*, *Cryptococcus*) also occur significantly more often compared to the normal population [60]. The reduced number of memory T cells is possibly the reason for the difficulties in controlling viral infections such as varicella-zoster or Epstein-Barr virus [61]. It is worth mentioning that, contrary to the former assumption of only immunodeficiency in STAT3 DN syndrome, recent reports on autoimmunity phenotypes have been published. Goel et al. identified a cohort with systemic lupus erythematosus (SLE)-like phenotype and particular renal involvement, but other STAT3 DN patients also showed characteristic increased expression of interferon-stimulated genes (ISGs), neutrophil extracellular trap (NET) formation, and anti-NET autoantibodies in the absence of autoimmunity phenotype [62]. Closer examination of the overlapping clinical manifestations of DN STAT3 and STAT1 GOF syndrome (Fig. 4) revealed STAT1 hyperactivation in STAT3 DN patient cells [63, 64]. Here, an activating cross-regulation of the STAT1 signaling pathway by reduced expression of SOCS3 in STAT3 DN with increased STAT1 downstream gene expression is assumed. The in part similar symptoms of STAT3 DN and STAT1 GOF such as autoimmunity, CMC, or vascular complications might be explained in this way (see below). Ex vivo treatment of patient cells with ruxolitinib, as already successfully used in the treatment of STAT1 GOF, effectively improved STAT1 overactivation [63, 65-70]. Therefore, it must be investigated in the future whether ruxolitinib is able to improve the symptoms in a disease model as well as patients (especially those with autoimmunity).

### Treatment

Since a curative therapy for HIES is not yet available, the most important goal at present is the intensive symptomatic treatment of infections, and even more so the prophylaxis of infections. Therefore, early diagnosis with analysis of the clinical phenotype by the application of the HIES score [27, 71] and subsequent genetic testing of possible genetic defects is essential. In addition to STAT3 and pSTAT3 levels, signs of STAT1 overactivation may also be investigated functionally [63]. Signs of a lack of classic inflammatory as well as autoimmunity symptoms should be considered, followed by a thorough evaluation of the patient’s medical history, current symptoms, and family history. Even a borderline negative HIES score should be further clarified, as there are isolated reports of young, unaffected carriers [34, 35]. Antibacterial and antifungal therapies should be initiated at an early stage to prevent the development of severe comorbidities and to keep survival and a high quality of life [52]. For this purpose, prophylactic antibiotics (e.g., trimethoprim-sulfamethoxazole against *S. aureus*) or topical antiseptics can also be used. Antifungal prophylaxis is indicated in recurrent or chronic candidiasis; likewise, anti-*Aspergillus* prophylaxis is indicated in predilected patients (e.g., with pneumatoceles) due to the increased risk of aspergillomas. As soon as lung contamination is present, it should be treated antifungally. Education in airway clearance techniques for patients with bronchiectasis is also important to prevent infections in the first place. If patients suffer from recurrent respiratory tract infections despite prophylaxis, immunoglobulins can be administered intravenously [72-74]. Preliminary in vitro studies on the potential therapeutic use of JAK inhibitors are already being conducted in patients with autoimmunity or autoinflammation symptoms associated with STAT1 overactivation. [63]. Considering that JAK inhibitors brought a significant reduction in STAT1 hyperactivation in experiments in vitro of AD-HIES patient cells, this might provide a previously unimagined therapeutic opportunity. Lobo et al. emphasize the need for preclinical work to explore on- and off-target effects of JAK inhibitors in AD-HIES patients as well as in vivo evaluation of efficacy and adverse effects. However, close surveillance should always be carried out, for example, to counteract the increased risk of fungal infection associated with JAK inhibitors at an early stage.

Because current therapies do not provide a causal solution but are limited to continuous antimicrobial prophylaxis, research is being conducted into other treatment options. Although allogenic peripheral stem cell transplantation did not seem to be effective when performed initially [75-77], major achievements have been made in the last few years: A recent study was able to show a reduction in the frequency of respiratory infections, skin diseases, and an improvement in lung function [78]. In addition, a reduction in IgE levels and, most notably, physiological populations of functional T_H_17 lymphocytes have been demonstrated. Although the results could only be shown in a small group of 14 patients, it can be postulated that allogeneic hematopoietic stem cell transplantation (HSCT) in principle can correct the immunodeficiency and patients thereby benefit clinically [78, 79]. However, no significant improvements in extra-hematopoietic symptoms could be observed [78, 80, 81]. In summary, HSCT offers great potential as a profitable therapy option. However, as only a few patients have undergone HSCT so far, the clinical relevance of serious complications such as graft versus host disease (GvHD) or vasculopathies remains unclear [82]. Although it has not yet been defined which criteria (e.g., major lung disease) have to be fulfilled in order to recommend HSCT treatment to patients with the best possible risk–benefit ratio, it is obvious that traditional infection prophylaxis therapy can be sufficient in the case of minor disease manifestations. Particularly in the case of early disease manifestation with a high risk of severe progression, transplantation should be considered as early as possible [79], because the transplantation outcome can be impaired by disease progression and the associated pulmonary damage itself as well as irreversible HIES complications such as lymphoma can be prevented. However, as not for every patient an HLA-matched donor can be found, gene therapy approaches are under development. The aim of these therapies is to genetically edit the patient’s own cells in a targeted and highly specific way ex vivo in order to subsequently use them in an autologous transplantation [83, 84]. It remains to be seen whether this new treatment option is curative and can be offered for a larger number of patients.

Overall, *STAT3* DN mutations can lead to autosomal dominant HIES with a characteristic immunodeficiency triad of elevated IgE, cyst-forming pneumonia, and recurrent cold skin abscesses caused by deficient STAT3-mediated T_H_17 differentiation. In addition to non-immunological symptoms, recent reports of autoimmunity expand the clinical picture. Autosomal recessive mutations in signaling elements influencing the JAK/STAT3 pathway, such as *ZNF341*, manifest with similar characteristics and can be poorly distinguished in clinical diagnostics with the HIES score. The focus is on prophylaxis to prevent life-threatening infections or chronic diseases and is complemented by causal therapy methods such as HSCT and future techniques.

## Heterozygous *STAT3* Gain-of-Function Mutations

### Overview

Heterozygous *STAT3* GOF germline mutations lead to an early childhood onset multiorgan autoimmune disorder (OMIM 615,952) known as infantile-onset multisystem autoimmune disease-1 (ADMIO1). It is characterized by a wide spectrum of clinical and immunological features which were first described in five unrelated patients in 2014 by Flanagan et al. [85]. Since then, more than 83 patients with over 49 different GOF variants of the *STAT3* gene have been described [86].

### Clinical Phenotype

A systematic review by Fabre et al. in 2019 provided great insight into characteristic symptoms of patients with STAT3 GOF mutations, which include immunological as well as systemic features [87]. More than half of the patients with *STAT3* GOF mutations present with autoimmune cytopenia (immune thrombocytopenic purpura (ITP), 24/42 described patients), autoimmune hemolytic anemia (AIHA, 19/28), and neutropenia (9/28). Other features are lymphoproliferation disorders such as lymphadenopathy (23/42), hepatosplenomegaly (23/42), Hodgkin’s lymphoma (1/42), or T cell large granular lymphocyte leukemia (T-LGL, 1/42) but also autoimmune-associated disorders like enteropathy (24/42), interstitial lung disease (15/42), hypothyroidism (13/42), and type I diabetes mellitus (10/42). In contrast to STAT3 DN syndrome, immunodeficiency is not always the salient feature of patients, although a large number of patients required immune system related therapy (see below). The majority of the patients showed an increased susceptibility to (myco-)bacterial, viral, and fungal infections, which mainly affected the lower and upper respiratory tract. The described symptoms can be complemented by postnatal growth failure, delayed onset of puberty, and cutaneous (e.g., atopic dermatitis), inflammatory (e.g., arthritis), musculoskeletal (e.g., osteoporosis, osteopenia, joint laxity, dental abnormalities), renal (e.g., nephrolithiasis), or ocular (e.g., uveitis) manifestations. The diversity of patients’ symptoms supports the suspicion of a STAT3 GOF syndrome [87].

The different phenotypes make precise and early diagnosis difficult. It is important to consider the patients in the context of all their symptoms, so that an experienced immunologist can make the correct diagnosis, especially in the presence of other symptoms that result in a STAT3 GOF syndrome. However, it should be noted that occasionally asymptomatic and only mildly affected patients have been described; hence, the incomplete penetrance should also be taken into consideration (see below) [86, 88-92].

### Genotype

Genetic testing of the *STAT3* gene locus in suspected congenital autoimmune disease, associated with other symptoms of STAT3 GOF syndrome, is currently the most valid diagnostic test as there are currently no established biomarker or pathognomonic immune cell alterations associated with *STAT3* GOF. Hence, only cloning of the variant and subsequent in vitro functional testing can provide information about the pathogenicity of variants that have not been previously identified [91]. Activating STAT3 germline mutations have been documented in the DNA binding domain, the SH_2_ domain, the transactivation domain, and the coiled-coil domain [88]. Most of the mutations occur de novo, but in approximately one quarter of the cases, the mutations are inherited in an autosomal dominant manner. With only few exceptions, there are exclusively missense mutations that lead to an amino acid exchange [88]. Interestingly, *STAT3* LOF missense mutations also occur at the same sites in the *STAT3* gene, so that the effect on STAT3 function seems to depend largely on the amino acid inserted and its charge (see below).

In addition, 15 unaffected *STAT3* GOF mutation carriers were identified in family studies [86]. The observation of phenotypic heterogeneity and incomplete penetrance in STAT3 GOF raises the question whether *STAT3* mutations alone may not be sufficient for disease onset, but that other, elusive factors must be present. Because of the variable phenotype of patients with *STAT3* GOF mutations and the different possible pathomolecular mechanisms of STAT3 hyperactivation, the question of genotype–phenotype correlation arises; however, no clear correlation could be demonstrated so far. However, it seems that mutations within the DNA binding domain present with a more severe phenotype. Further case reports and systematic reviews may contribute significantly to the elucidation of this question, thus advancing patient-centered medicine. Why STAT3 GOF and STAT3 DN present with different penetrance patterns remains unknown. It is plausible that the LOF phenotype may be less broad than the GOF phenotype what might be one major distinguishing feature of *STAT* LOF and *STAT* GOF mutations. The influence of hyperactivated STAT3 molecules at different pathway levels is manifold (e.g., basal transcription, DNA binding affinity, phosphorylation and dephosphorylation kinetics, interaction with other pathways, T_reg_ defect). Thus, one can imagine many more ways in which activating mutations can dysregulate a signaling pathway.

### Molecular and Cellular Phenotype

Generally, amino acid substitution in the DNA binding domain was shown to increase DNA binding affinity and to lead to enhanced transactivation due to changes in the electrical charge of the STAT3 molecule [88, 93]; mutations in the SH_2_ domain were shown to lead to increased sensitivity to cytokines and enhanced dimerization [91]; mutations in the coiled-coil domain were shown to affect subcellular localization of the STAT3 dimer and increase nuclear import [91, 94]. Only occasionally, increased phosphorylated STAT3 (pSTAT3) levels are detected in unstimulated cells (such as in *STAT1* GOF mutations, see below [91, 95, 96]), and STAT3 expression levels were also normal, except for one case [96]. The overactivity of STAT3 can be explained by an increased intrinsic transcriptional activity of the STAT3 dimer rather than by enhanced expression or enhanced phosphorylation although cases of delayed dephosphorylation have been described [88, 97]. In 2020, Jägle et al. examined 17 different *STAT3* GOF variants with regard to their molecular behavior [91]. They were able to establish correlations between the molecular response pattern and the clinical phenotype. Based on their findings, they derived three distinct GOF groups: Group 1 is characterized by significantly increased basal transcriptional activity, increased DNA binding affinity, delayed dephosphorylation, and furthermore increased STAT3 phosphorylation after IL-6 stimulation. In this group, the mutations were only located in the coiled-coil domain, which may lead to delayed dephosphorylation via more efficient receptor binding or reduced interaction with cytoplasmic phosphatases. It seems reasonable to explain the very strong disease penetrance compared to the other groups by strongly increased, stimulus-independent basal transcription activity. In group 2, basal transcriptional activity is only slightly increased, but can increase significantly upon stimulation; clinical penetrance is correspondingly lower than in group 1. Penetrance in group 3 was moderately increased, and the basal transcription rate was normal; a prolonged phosphorylation, increased nuclear pSTAT3 accumulation, and DNA binding could be detected. While the group of Jägle et al. cannot comprehensively elucidate the phenotypic differences of the patients, it does provide evidence for incomplete penetrance via the heterogeneous molecular mechanisms. The classification of *STAT3* mutations into functional groups now appears biologically and clinically meaningful. However, the authors point to other hitherto unknown factors such as additional mutations, epigenetic changes, crosstalk with other pathways, or environmental factors (especially infections), which probably also influence penetrance.

From an immunological perspective, patients with *STAT3* GOF mutations are characterized by several hallmarks: More than half of the patients present with hypogammaglobulinemia and more than a quarter with NK cell lymphopenia, T cell lymphopenia, or B cell lymphopenia. In particular, regulatory T cells (T_regs_) and switched memory B cells are decreased, although these numbers may vary widely between patients [87]. STAT3, which is an IL-6 mediating transcription factor, has an essential influence on the differentiation and expansion of T_H_17 cells, a cell population known to induce autoimmunity when overactivated [98]. *STAT3* mutations are therefore predisposed to autoimmunity via overactivation. More generally, autoimmunity is thought to arise from a dysregulation of the equilibrium between T_H_17 and T_reg_ cells [98-103]. The low T_reg_ numbers observed in STAT3 GOF are consistent with the role of STAT3 in mediating forkhead box protein 3 (FOXP3)-inhibiting cytokines and concomitant inhibition of differentiation of naïve T cells into autoimmunity-controlling T_reg_ cells [104]. STAT3-dependent cytokines such as IL-6, IL-21, and IL-27 can inhibit FOXP3 expression by enabling STAT3 to bind to a binding site in the FOXP3 enhancer II acting as a silencer for FOXP3 transcription [105]. The effects on the instability of natural T_reg_ cells, as well as the inhibition of induced T_reg_ (iT_reg_) cell polarization from I CD4^+^ T cells, are relevant [106]. However, due to increased expression of STAT3 target genes, the negative regulator SOCS3 is also produced at a higher levels limiting not only STAT3 but also indirectly disrupt STAT5b in its function [88, 107, 108]. In healthy individuals, STAT3 and STAT5 possess opposing regulatory mechanisms essential for the existence of functioning T_reg_ cells [109]. It is assumed that some symptoms can therefore be explained by a reduced function of STAT5b. This is supported by overlapping symptoms of patients with *STAT3* GOF and *STAT5B* LOF mutations, like cytopenia, short stature, and enteropathy [110]. The attenuation of STAT5b leads to impaired development of T_reg_ cells as a result of decreased CD25 and FOXP3 expression and reduced IL-2 signaling [111-113]. Impaired IL-2/CD25 signaling in defective T_reg_ cells may explain autoimmunity processes via the lack of negative regulation of T_H_17 cells [112, 113]. Fabre et al. hypothesized that in most cases, patients’ T_H_17 counts have been affected by immunosuppressive therapy [87]. Therefore, a more detailed study of T_H_17 development in untreated STAT3 GOF patients would be beneficial. Since STAT3 GOF and immunodysregulation polyendocrinopathy enteropathy X-linked (IPEX) syndrome share symptoms such as autoimmunity, together with reduced T_reg_ cell frequency, it is arguable that STAT3 GOF belongs to the spectrum of “Tregopathies” [114]. A very recent mouse model study could not provide evidence that T_reg_ defects are major drivers of STAT3 GOF syndrome, but collect evidence for selective deficiency in generation of iT_reg_ cells and an expansion of effector CD8^+^ T cell population [115]. In this context, it is worth mentioning the SOCS1 haploinsufficiency, first described in 2020, which presents with symptoms that are in part similar to those of STAT3 GOF (lymphadenopathy and autoimmune cytopenia) [116]. In this disease entity, which affects the STAT1- and STAT3-regulating negative modulator, an increased JAK/STAT pathway activation after stimulation with IFN-γ, IL-2, and IL-4, T cell hyperproliferation in response to IL-2, and a reduced T_reg_ frequency and suppressive activity are found. This study provides further evidence for the role of the T_reg_ compartment in the development of autoimmunity by revealing cytokine hypersensitivity through deficient negative regulation of STATs. It should be noted that compared to STAT3 GOF, there was incomplete penetrance but also differences in manifestation (SLE in SOCS1 haploinsufficiency vs. enteropathy and diabetes in STAT3 GOF).

### Treatment

Treatment of patients with *STAT3* GOF mutations was initially limited to immunosuppressive and immune-modulatory therapy. In most cases, patients receive steroids, mycophenolate mofetil, tacrolimus, rituximab, sirolimus, and anti-TNF-α antibodies. Respiratory bacterial infections can be prevented or treated with antibiotics, and treatment can be supplemented with respiratory physiotherapy. Intravenous or subcutaneous immunoglobulin substitution may help patients with hypogammaglobulinemia [92]. However, by understanding the pathomolecular underlying STAT3 hyperactivation, it is now possible to offer patients targeted therapies. For example, the monoclonal anti-IL6-R antibody tocilizumab, which inhibits the upstream of STAT3 signaling cascade, is already being used with good success [68]. In combination with - in cancer therapy successfully tested - JAK inhibitors ruxolitinib (JAK1/2 inhibitor) and tofacitinib (JAK1/3 inhibitor), additional treatment success with resolution of enteropathy and respiratory function was achieved in five of eight patients. In contrast to *STAT1* GOF mutations, HSCT so far did not reveal a good outcome: only in two out of six treated patients, HSCT could meet the objective of a causal curative treatment with complete resolution of autoimmune manifestations [88, 117]. In the other four cases, fatal complications occurred due to GvHD, systemic adenoviral infection, or hemophagocytic lymphohistiocytosis [68, 108, 118]. The reported numbers are still too small to judge if HSCT is a suitable therapeutic option for patients with STAT3 GOF, and more research regarding predictors of outcome is needed to recommend this treatment option. New drugs targeting the STAT3 pathway are in development, but only few are already investigated in clinical trials [119, 120]. Interesting and promising approaches in vitro are small molecule STAT3 inhibitors that disrupt STAT3 dimerization or promote STAT3 protein degradation [121-123]. Additionally, research is being conducted on oligodeoxynucleotide (ODN) decoys which act competitively as DNA binding domain inhibitors to block the binding of STAT dimers to endogenous STAT-responsive elements in promoters of STAT-regulated genes, and antisense oligonucleotides (ASOs) that interfere with *STAT3* mRNA and downregulate STAT3 expression [95, 119]. Here, both immunology and cancer research can benefit from each other.

### STAT3 and Cancer

Since the connection between inflammation and tumor development, for example, through inflammation-induced initiating of oncogenic transformation by recruiting immune cells, has become known, attention in tumor research has also fallen on STAT3 [124-126]. STAT3 plays a major role in biological processes such as cell proliferation, survival, differentiation, and angiogenesis, and it has been shown that STAT3 overactivation in tumors (e.g., through a loss of negative regulatory mechanisms, excessive cytokine stimulation or activation of positive feedback loops, rarely *STAT3* GOF mutations) is often associated with a worse prognosis and accelerated disease progression [127, 128]. Somatic *STAT3* GOF mutations can be detected in 30–40% of T cell large granuloma lymphocytic (LGL) leukemia and chronic lymphoproliferative disorder of natural killer cells (CLPD-NK) [129-132]. Both are phenotypically similar, morphologically different subtypes that may cause a clonal expansion of the respective cell type (e.g., CD3^+^ large granular lymphocytes in T-LGL) [133, 134]. The patients’ clinical phenotype is defined by recurrent infections, anemia, neutropenia, splenomegaly, and autoimmune diseases, in particular rheumatoid arthritis [135]. The mutation was consistently found in the SH_2_ domain, resulting in increased dimerization, activation of the STAT3 protein, and transcriptional activation [132, 136]. However, STAT3 has also been attributed tumor suppressor properties as seen in studies with *STAT3*-deficient mouse models. They suggest that it may act as a negative regulator in certain tumor entities (e.g., prostate cancer [137], glioblastoma [138], intestinal cancer [139], lung cancer [140], and thyroid cancer [141]).

## Biallelic and Heterozygous *STAT1* Loss-of-Function Mutations

### Overview

Mutations in *STAT1* may cause autosomal dominant or autosomal recessive diseases. STAT1 is heavily involved in mediating cytokine signaling of type I (IFN-α/β), type II (IFN-γ), and type III (IFN-λ) interferon as well as IL-6, IL-9, IL-10, IL-11, IL-12, IL-19, IL-21, IL-27, epidermal growth factor (EGF), platelet-derived growth factor (PDGF), ciliary neurotrophic factor (CNTF), LIF, and OSM [142, 143]. IFN-α/β, IFN- γ, and IL-27 are known as typical cytokines that hinder the development of T_H_17 cells producing IL-17A, IL-17F, and IL-22. *STAT1* has been described to have activating GOF but also LOF mutations.

As STAT1 is central for the integrity of the immune system, *STAT1* LOF mutations can also affect the patient’s immune responses in a characteristic manner. The defects that occur can be classified into three categories: autosomal recessive complete STAT1 deficiency, autosomal recessive partial STAT1 deficiency, and autosomal dominant STAT1 deficiency [144-147]. The severity of the clinical picture depends on the severity of the defect and the ability of the cell to mediate certain extracellular signals via STAT1.

### Genotype

Patients with autosomal recessive complete STAT1 deficiency completely lack STAT1 expression and therefore the ability of pSTAT1 induction after IFN type I, II, and III as well as interleukin stimulation (e.g., IL-27). Only 24 patients with an autosomal recessive complete STAT1 deficiency (16 different mutations) have been described and 17 died, most likely because of its profound consequences for the immune system (see below). Interestingly, in 2020, Sakata et al. described the first intronic mutation in the *STAT1* gene causing autosomal recessive complete STAT1 deficiency [148]. Since intronic mutations may also occur, the genetic diagnosis may be challenging due to variants of unknown significance in intronic regions. In order to identify whether individual variants have pathogenic potential, whole genome sequencing should be followed by targeted RNA sequencing or *STAT1* mRNA analysis. These can reveal potential STAT1 expression reductions caused, for example, by mutations in splicing regulators leading to aberrant pre-mRNA splicing and consecutive protein degradation. Protein analysis and STAT1 functional testing can follow to assess the final impact of an intronic variant on protein level.

### Clinical Phenotype

Patients with these *STAT1* null mutations suffer from extensive infections of intracellular bacteria (especially mycobacteria) and viruses (typically herpes viruses) as well as in some cases secondary hemophagocytic syndrome. Most of these complications occur after a few months and may be lethal in the first two years of life. A *STAT1* LOF mutation must be considered when evaluating patients with the clinical picture of severe mycobacterial and viral infections.

### Molecular and Cellular Phenotype

Pathomolecularly, the lack of a STAT1-mediated response to IFN-γ, IFN-α/β, IFN-λ, and IL-27 is the cause of an inadequate activation of effector cells, which are supposed to fight off the actually weakly virulent pathogens (e.g., *Mycobacterium kansasii*, HSV-1, CMV) [149].

### Treatment

Out of 24 published patients, ten patients received HSCT of whom only seven patients are alive [147, 150-152]. HSCT is currently the only therapeutic option for this condition.

### Partial autosomal recessive STAT1 deficiency

In addition, patients with a milder form of STAT1 LOF have been reported [153]. Both the milder, “partial” variant and the complete STAT1 LOF result from biallelic mutations. However, the partial deficiency does not lead to a total loss-of-expression or loss-of-function, but rather to a reduced production and attenuated function of the STAT1 protein [144]. As can be assumed, the ability of the patient’s cells defending viral (especially herpes viruses) and intracellular bacterial (especially mycobacteria) pathogens is impaired, but not lost. The intensity of the immune response and thus the patient’s clinical condition depends on the mutation and the level of remaining STAT1 activation. Obviously, the disease is less severe in patients with partial STAT1 LOF than in patients with complete STAT1 LOF, so that consequent antibiotic/antiviral therapy improves the outcome of those affected.

### Autosomal dominant STAT1 deficiency

Since 2001, 31 patients with autosomal dominant STAT1 deficiency have been documented [144, 154]. They show heterozygous mutations in the DNA binding domain [155], the SH_2_ domain [156, 157], or the tyrosine residue Y701 [158], which is mainly responsible for the activation of the protein through phosphorylation. In contrast to the groups described above, these patients suffer solely from mycobacterial infections with weakly pathogenic mycobacteria, which are usually relatively mild [159, 160]. The DN *STAT1* alleles do not lead to a predisposition to viral infections, which can be explained by an unaffected response to IFN-α/β in heterozygous cells. The IFN-γ response, on the other hand, appears to be limited by a heterozygous mutation [144, 159-161]. The attenuated IFN-γ response is mainly responsible for the defense against intracellular bacteria and thus the pathomolecular cause of the patients’ infections [144].

## Heterozygous *STAT1* Gain-of-Function Mutations

### Overview

As can be seen, too little STAT1 immunity (from biallelic or DN mutations) is associated with a susceptibility to mycobacterial and viral infections. A balanced equilibrium of STAT1 signaling is central to avoid severe and possibly life-threatening infections, because STAT1 GOF typically leads to autoimmunity and CMC. Since its first description in 2011 by Van de Veerdonk et al., the STAT1 GOF phenotype, characterized by immunodeficiency and autoimmunity, has been well described [162].

### Clinical Phenotype

The most prominent symptom of patients is the presence of CMC, which can be observed in about 90% of all patients before the age of 10 years (early-onset CMC), yet the lifetime risk of CMC is nearly 100% [162, 163]. The main pathogen causing mucocutaneous infections is *Candida albicans*, although invasive infections with *Candida albicans*, *Cryptococcus* spp., *Pneumocystis jirovecii*, *Aspergillus* spp., and *Penicillium marneffei* can also be observed in approximately 20% of cases. Overall, around half of all CMC cases appear to be attributable to *STAT1* GOF mutations [164-166], highlighting the importance of CMC as a diagnostic criterion for *STAT1* GOF mutation. In addition to fungal pathogens, patients with *STAT1* GOF mutations are susceptible to recurrent lower respiratory tract bacterial infections (LRTI). Infections with *S. aureus*, *Streptococcus* spp., *Pseudomonas aeruginosa*, or *H. influenzae* can - in some circumstances - lead to long-lasting LRTI that can result in severe pneumonia, bronchitis, interstitial pneumonia, or more persistently, bronchiectasis. Mycobacterial diseases may occur after infections of tuberculous (i.e., *Mycobacterium tuberculosis*) or non-tuberculous mycobacteria (i.e., *Mycobacterium avium)* as well as BCG vaccine, including tuberculosis, skin disease, and adenitis [163]. Viral infections with *Herpesviridae* (herpes simplex virus, varicella-zoster virus, *Cytomegalovirus*, Epstein-Barr virus) have also been commonly described [163]. On the basis of immune dysregulation, patients may develop an IPEX-like autoimmune phenotype, which may present as autoimmune thyroid disease, autoimmune cytopenia, vitiligo, psoriasis, alopecia, type I diabetes, autoimmune hepatitis, enteropathy, systemic lupus erythematosus, or polyendocrinopathy. *STAT1* GOF mutations are associated with an increased incidence of certain tumor entities, some of which may be explained by persistent mucocutaneous fungal infections that develop into squamous cell carcinoma (cutaneous, oral, laryngeal, esophageal, gastrointestinal cancer). STAT1 GOF patients also present with melanoma, lymphoma, leukemia, prostate cancer, or papillary thyroid cancer [163]. Aneurysms occur three times more frequently than in the normal population (10.7%) and may remain asymptomatic but might burst, especially intracerebrally [163, 167]. Chronic (fungal) infections with the development of drug resistance, malignancies, and aneurysms associated with hemorrhages (particularly in the brain) are predictors of poor outcome in STAT1 GOF patients [164].

### Cellular Phenotype

The symptoms of the patients can be partly explained by the changes in immune cell subpopulations. Eighty-seven percent of patients with *STAT1* GOF mutations have a deficiency of T_H_17 cells, which is due to a poor development of IL-17A-, IL-17F-, and IL-22-producing cells [164]. Increased responses to IFN-α/β, IFN-γ, and IL-27, which are all STAT1-dependent repressors of IL-17 producing cells [142, 168-172], hinder the development of those cells in mice and humans. Interestingly, the lack of T_H_17 cells, which are necessary for preventing CMC, cannot be observed in all patients (82%), whereas 98% of patients develop CMC [164, 173-184]. Therefore, other pathomechanisms of immune dysregulation might be present (see below). Last but not least, the very high prevalence of CMC (98%) in STAT1 GOF should also be considered to be due to ascertainment bias, where, e.g., other dermatological manifestations such as eczematous dermatitis are misinterpreted as CMC, which could overestimate the prevalence of CMC in STAT1 GOF patients. This would help to understand why not all patients diagnosed with CMC have reduced T_H_17 counts. There are individual case reports showing impaired production of IL-6, IL-17A, and IL-22 and increased production of IL-4 in comparison to healthy individuals [185, 186]. Interestingly, IL-4 can suppress T_H_17 cell-mediated immunity via IL-23 silencing [187]. Decreased IFN-γ responses and increased IL-4 production, as typically found in atopic diseases [188], cannot be detected in the majority of STAT1 GOF patients to a clustered extent. Nevertheless, STAT1 GOF has been attributed an atopic phenotype because it can manifest as an IPEX-like syndrome with the above-mentioned autoimmune manifestations, enteropathy, and eczematous dermatitis [189-191]. Because no IPEX-typical reduction of T_reg_ cells or *FOXP3* mutations could be found in STAT1 GOF, another mechanism must underlie this phenotype (see below) [191]. Other lymphocyte subpopulations are also reduced: diminished numbers of CD4^+^ and CD8^+^ T cells are observed (23% of patients have low T cells, 30.8% have low CD4^+^ cells, 17.9% have CD8^+^ low, and in some cases both CD4^+^ and CD8^+^ are reduced), CD19^+^ or CD20^+^ B cell numbers are low in 23.9%, and most patients have reduced numbers of CD19^+^CD27^+^ memory B cells. CD16^+^CD56^+^ NK cells are low in 32% and show a reduced NK cell toxicity. In addition, humoral immunodeficiency with low serum IgG, IgA, or IgM levels was described in 6.5%, 18.3%, and 6.3% of patients, respectively [163]. Elevated serum IgG, IgA, or IgE levels were described in 20%, 4%, and 3% of patients, respectively [164].

### Genotype

The underlying genetic causes for STAT1 GOF are activating mutations in mostly the coiled-coil domain but also in the DNA binding domain, linker domain [192], or SH_2_ domain [193]. More than 110 missense mutations and one deletion have been described [163].

### Molecular Phenotype

The resulting mechanisms for hyperactivation of STAT1 are the subject of current research and are not yet fully understood in their complexity. Various, sometimes opposing, hypotheses exist for the increased molecular activity of the STAT1 protein. Easily understood are results that imply an impaired cycle of phosphorylation or dephosphorylation, where either the phosphorylation of STAT1 occurs faster or the nuclear dephosphorylation is slowed down [142]. In experiments with the protein kinase inhibitor staurosporine, a persistently high level of pSTAT1 was observed, supporting the latter hypothesis. This is opposed by findings that observed a normal or even faster rate of dephosphorylation in CD14^+^ monocytes with concomitant high levels of total STAT1 expression [65, 192, 194-196]. These findings are complemented by the observation of high *STAT1* mRNA levels [194, 197, 198]. Other studies suggest premature nuclear import with normal phosphorylation/dephosphorylation rate [168, 199], increased nuclear accumulation (*STAT1*^R247W^), decreased mobility (*STAT1*^R321S^, *STAT1*^N571I^), or immobility in the nucleus (*STAT1*^T419R^) as the cause of STAT1 hyperactivation [200]. There also exists the concept that DNA binding specificity decreases with certain *STAT1* GOF mutations as fewer IFN-γ activation site (GAS) motifs are detectable in promoters of STAT1 GOF-specific genes. The ratio of GAS present to GAS absent in promoters of STAT1-regulated genes decreased significantly for the *STAT1*^T419R^ variant in chromatin immunoprecipitation (ChIP)-Seq experiments [170]. In RNA-Seq analyses, dysregulated gene transcription caused by the *STAT1* variants resulted in different mutation-specific fingerprints and offers a possible explanation for the wide range of phenotypic variation. Reasonably well described is the observation that GOF mutations in the coiled-coil domain and DNA binding domain impair the stabilization of the STAT1 homodimer in an antiparallel conformation. Physiologically, conversion from parallel conformation to antiparallel conformation is required for dephosphorylation at pY701 by phosphatases. By remaining in the parallel conformation and lacking access to pY701, STAT1 GOF homodimers remain phosphorylated and become resistant to dephosphorylation [201, 202].

Finally, the different mechanisms may all lead to a hyperactivation of STAT1. Increased responses to IFN-α/β, IFN-γ, and IL-27 by STAT1 hyperactivation may explain the impaired development of T_H_17 cells since those cytokines are STAT1-dependent repressors for T_H_17 differentiation in mice and humans [173-184]. In addition, in patient cells with *STAT1* GOF mutations, enhanced IL-27 signaling upregulates the expression of programmed death-ligand 1 (PD-L1) [64, 203]. As PD-L1 is known suppress T_H_17 differentiation, this process may contribute to defect induction of mucosal immunity against fungal pathogens [204, 205]. Based on its hyperactivity and the fact that STAT1 can sequester STAT3 in non-functional heterodimers [206-209], it would also be conceivable that an increased STAT1 response inhibits STAT3 by utilizing the process of heterodimerization. Our group is investigating the influence of *STAT1* and *STAT3* mutations, respectively, on the process of heterodimerization, and how different mutations affect STAT1:STAT3 heterodimer formation. As STAT3 is the key transcription factor for T_H_17 development (see above), this concept provides an explanation for the high prevalence of CMC in both patients with STAT3 LOF mutations and patients with STAT1 GOF mutations. The negative effect of STAT1 GOF on STAT3-driven T_H_17 cell induction is the most likely reason behind the overlapping symptoms of both syndromes (Fig. 4). Independent of heterodimerization, a negative effect of STAT1 GOF on STAT3 transcriptional activity was detected in ChIP assays, resulting from altered STAT3 promoter binding most likely due to reduced histone acetylation through STAT1 [208]. Thus, it appears that *STAT1* GOF mutations can have an influence on an epigenetic level. Furthermore, Kaleviste et al. showed a significant enrichment of H3K4me3, a marker for activation of gene transcription by chromatin remodeling, in interferon-stimulated genes in patient cells compared to healthy donors, even in the absence of type I interferons [193]. These observations promote the idea that the increased IFN signature of patients with STAT1 GOF might be epigenetically fixed. As patients with STAT1 GOF and type I interferonopathies show overlapping clinical features in autoimmunity, predisposition to IFN-related autoimmunity seems plausible and might need to be taken into account when deciding for personalized therapy [167, 210].

STAT1 GOF is one cause of an IPEX-like phenotype, hence IPEX-typical symptoms of primarily normal T_reg_ cell number and function including *FOXP3* wild type. LOF mutations in *CD25*, *STAT5B*, and *ITCH* were identified as other causes, which can be differentiated from each other by specific symptoms. Since FOXP3-dependent T_reg_ development does not appear to be defective, the central functions of the IPEX-like associated proteins for T_reg_ function suggest an immune dysregulation of the same [190]. For example, STAT5 mediates IL-2 signaling via the receptor CD25 resulting in the production of anti-inflammatory IL-10 and generation of induced T_reg_ cells [211, 212]. The IPEX-like phenotype is associated with enhanced DNA binding and transactivation and prolonged STAT1 phosphorylation after IFN-γ, IFN-α, IL-6, and IL-21 stimulation [191, 213, 214]. This STAT1 overactivation, as it also occurs for example in recombinant IFN-α therapy as a phenocopy of STAT1 GOF, seems to be involved in the pathogenesis of autoimmunity [215-217]. This is supported by the observations of severe inflammation and autoimmunity in type I interferonopathies that feature upregulation of type I interferon signaling [210, 218, 219]. It should also be noted that *STAT1* GOF, *STAT3* LOF, *IL-10* LOF, and *IL21R* LOF mutations can alter circulating T_FH_ (cT_FH_) cell differentiation by producing fewer total cells or different cT_FH_ cell subsets [220]. The resulting populations showed a phenotype with overexpression of IFN-γ but also deficient induction of humoral immunity, consistent with the impaired humoral immune response in STAT1 GOF and STAT3 LOF [203, 221]. The overexpression of IFN-γ points again to autoimmunity as it occurs in many diseases such as multiple sclerosis and rheumatoid arthritis. In 2013, Uzel et al. found no abnormalities in T_reg_ counts and functional tests, but the latter were limited to only two patients [191]. Even in a larger IPEX(-like) cohort, only a non-significant tendency toward a lower T_reg_ frequency compared to controls could be detected [222]. Moreover, the suppressive function in the presence of STAT1 signaling cytokines and the resistance of responder cells or APCs to T_reg_-mediated suppression in particular were not investigated [191]. Accordingly, the pathomechanism of IPEX-like disease in STAT1 GOF remains unclear over several years. Instead, there is speculation about *STAT1* mutants destabilizing the T_reg_ cells by reprogramming them into T_H_1-like cells, altering transcriptomes, and producing functional IL-10 deficiencies [190, 223]. Interestingly, inhibited T_reg_ cell development and altered STAT1-mediated gene transcription were detected in a STAT1 GOF patient with common variable immunodeficiency (CVID) symptoms - but without classical IPEX-like symptoms [224]. Pathomechanistically, this is due to instability of the antiparallel STAT1 dimer conformation, such that the dephosphorylation of parallel dimer conformation is slowed down, resulting in an increased induction of the IFN-target genes. Various publications point out a significant negative influence of STAT1 on the development of T_reg_ cells, in particular by collecting evidence for increased T_reg_ expansion in the absence of STAT1 [225-228].

Similar to *STAT3* GOF mutations, STAT1-induced SOCS1-mediated suppression of STAT5 may occur with *STAT1* GOF mutations. Since STAT5 is known to be involved in terminal NK cell differentiation, NK cell toxicity, and perforin expression, this may explain increased viral susceptibility in patients with *STAT1* GOF mutations [197, 229, 230]. Likewise, negative regulation of STAT3 via increased SOCS1 production in STAT1 hyperactivation would be also plausible [64].

### Diagnostics

In light of the characteristic symptoms, treating physicians should consider the differential diagnosis STAT1 GOF in patients with CMC alone, CMC with lower respiratory tract infections, or CMC with autoimmune thyroid disease. Altered lymphocyte subpopulations can be determined by lymphopanel analyses. In this context, various genetic and functional tests are useful for strengthening the diagnosis of STAT1 GOF, e.g., subcellular distribution or levels of STAT1, pSTAT1, interferon-stimulated gene factor 3, and γ-activated factor (GAF) in unstimulated and in IFN-α-, IFN-γ-, or IL-27-stimulated conditions. Also, gene transcription induced by interferons or in vitro luciferase STAT1 reporter assays may be helpful for diagnosing STAT1 GOF. Eventually, DNA sequencing can provide information about the exact genotypic variation of the patient. Integrating such findings could serve to identify possible genotype–phenotype correlations. According to Zhang et al. the median onset of symptoms in STAT1 GOF patients is 1 year, but the median diagnosis does not occur until 6.2 years, implying a noteworthy delay of diagnosis [163].

### Treatment

The treatment of patients with *STAT1* GOF mutations has advanced considerably over the past 10 years. While the initial focus has been on symptomatic treatment of patients with abatement of infection-related symptoms, treatment options nowadays aim at a more causal approach. Topical or systemic antifungal therapy (preferably triazoles such as fluconazole), prophylactic or ad hoc antibiotic therapy, and immunoglobulin replacement therapy especially for the prophylaxis or treatment of pneumonia is commonly used. Eczematous dermatitis may be treated with topical steroids or topical application of calcineurin inhibitors.

With the advent of JAK inhibitors (jakinibs) such as ruxolitinib and baricitinib (both inhibiting predominantly JAK1 and JAK2), targeted therapeutic options are available for STAT1 GOF patients and have improved symptoms to even resolution of CMC in 12/20 patients [65-68, 70, 229]. However, due to the possibility of varicella-zoster virus and *Cytomegalovirus* (VZV and CMV) exacerbation due to higher risk during JAK inhibitor treatment and the already increased risk of infections in patients with *STAT1* GOF mutation, a prophylactic treatment with aciclovir should be considered [231, 232]. The exact risk remains unclear since besides the adverse effects, a positive effect on NK cell function has also been shown in studies [229]. Antifungal prophylaxis against worsening of fungal infections observed in several patients treated with ruxolitinib, such as CMC and coccidioidomycosis (especially dusty areas in southwestern USA, Mexico, and South America), should also be discussed [68, 192].

In recent studies, ruxolitinib was considered a useful drug for bridging therapy in patients with *STAT1* GOF mutations waiting for HSCT to reduce post-transplant complications [230]. Approximately 40% of STAT1 GOF patients have died following HSCT due to secondary graft failure, or gastrointestinal or pulmonary bleedings [163]. Ruxolitinib is considered to reduce the enhanced IFN signaling in blood and tissue cells and therefore the immune dysregulation caused by *STAT1* GOF mutations. Mechanistically, this could help to downregulate uncontrolled inflammation and therefore minimize the risk of secondary graft failure. The purpose of the concomitant and bridging therapy is to restore the STAT1-mediated immune response to a normal state until HSCT can correct the underlying genetic defect. The studied patient showed stabilization by therapy with ruxolitinib (improved STAT1 phosphorylation/dephosphorylation, normalized number and response of T_FH_ cells, partly improved health status and IFN signature) but persistent T_H_17 deficiency. Subsequent HSCT normalized STAT1 phosphorylation/dephosphorylation, dysregulated gene expression, the IFN signature, and T, B, and NK cell function. Further studies are necessary to better understand the challenging problems in transplanting patients with GOF mutations in which - at least theoretically - full conditioning and a 100% donor chimerism need to be achieved for a sustained therapeutic success.

## Heterozygous *STAT6* Gain-of-Function Mutations

### Overview

STAT6 is involved in the signaling pathways of several cytokines such as IL-3, IL-15, IFN-α, and PDGF, but plays a key role in the signal transduction of IL-4 and IL-13. IL-4 and IL-13 are significantly relevant to T_H_2 differentiation, B cell proliferation, IgE class switch, as well as generation and activation of eosinophil granulocytes and mast cells. Consequently, STAT6 is involved in the pathophysiology of atopic diseases such as allergy, asthma, or atopic dermatitis. While somatic STAT6 polymorphisms are mainly associated with various tumor entities such as Hodgkin lymphoma, only recently patients with germline *STAT6* GOF disorders have been reported [233, 234]. A germline STAT6 LOF phenotype has not yet been described in humans.

### Genotype

In 2022 and 2023, 21 patients from 13 unrelated kindreds with germline heterozygous *STAT6* GOF mutations were identified [234-237]. The missense variants mostly occurred de novo, though in four families, an autosomal dominant inheritance pattern has been reported. Complete penetrance of the disease was seen, with some clinical heterogeneity. The mutations are localized in the DNA binding domain, the linker domain, the SH_2_ domain, and near the phosphorylation site Y641 in the transactivation domain. Sharma et al. showed that all variants are located near the protein-DNA interaction region or in the STAT6-specific protein-DNA recognition site [237, 238]. They were able to show that almost all amino acid substitutions resulted in an increased electropositivity of the STAT6 protein.

### Clinical Phenotype

STAT6 GOF is a multisystem disorder characterized by its key symptoms early-onset multiple severe food allergic disease and severe treatment-resistant atopic dermatitis [234-237]. The phenotype always presents in the first year of life and may also include asthma, gastrointestinal/skin eosinophilic infiltration, or severe anaphylactic episodes. Furthermore, recurrent skin and respiratory infections of bacterial, fungal, and viral origin, failure to thrive, skeletal hypermobility, pathologic fractures, or vascular anomalies may occur [237]. In one patient, follicular and diffuse large B cell lymphoma have been described, with B cell lymphoma already known to arise from somatic *STAT6* GOF mutations [237, 239, 240]. In two cases, patients died, respectively, from anaphylaxis and subarachnoid hemorrhage secondary to cerebral aneurysms. Given the distinct atopic manifestations, STAT6 GOF is classified as a primary atopic disorder [237, 241]

### Molecular and Cellular Phenotype

The *STAT6* GOF variants studied showed increased pSTAT6 levels before and after stimulation compared to healthy controls, and there are reports of physiological but also slowed dephosphorylation kinetics [235, 237]. Suratannon et al. further observed enhanced nuclear accumulation of a *STAT6* variant [236]. Through increased electropositivity upon amino acid exchange and correspondingly enhanced interaction with negatively charged DNA (see above), the main mechanism of increased STAT6 activity seems to be increased DNA binding affinity. The investigated *STAT6* variants showed increased transcriptional activity in luciferase reporter assays in both the absence and presence of IL-4 [234, 237]. Transcriptomic analysis identified the increase of STAT6 target genes, including *IL4R*, *CISH*, and *XBP1* (involved in plasma cell and eosinophil differentiation), as well as an increase of T_H_2-inducing factors. For variants E382Q and D419G, partly unique transcriptional profiles were observed that are not known in wild type suggesting more GOF mechanism than only enhanced DNA binding. Patients typically show normal T, B, and NK cell counts, with high levels of T_H_2 lymphocytes next to signs of chronic inflammation [236]. The T_H_2 cytokines IL-4, IL-5, and IL-13, which are major contributors to the pathogenesis of atopic diseases, were significantly elevated in T_H_2 patient cells. T_H_2 skewing as well as suppressed T_H_1 and T_H_17 responses were evident in STAT6 GOF patients [235, 237]. Furthermore, increased IL-4Rα expression was detected in naïve and memory CD4^+^ T cells as well as non-class switched and class switched memory B cells. Consistent with the physiological function of STAT6, high serum IgE levels and hypereosinophilia were key features of STAT6 GOF patients.

In vitro, normalization of STAT6 activation by IL-4/STAT6 axis inhibitors ruxolitinib, tofacitinib, and the anti-IL-4Rα antibody dupilumab could be demonstrated [237]. In accordance with the clinical phenotype, in STAT6^D419N^ knock-in mice spontaneous development of dermatitis with skin thickening, eosinophilic skin infiltration, elevated serum IgE levels, and mRNA expression of type 2 cytokines could also be observed [234].

### Diagnostics

Sharma et al. propose a set of clinical findings for which the presence of STAT6 GOF should be further investigated [237]. These include early life symptom onset, peripheral blood eosinophilia, elevated serum IgE, treatment-resistant atopic dermatitis, multiple food and drug allergies, severe/fatal anaphylaxis, recurrent skin and respiratory infections, and eosinophilic gastrointestinal disorder including eosinophilic esophagitis, asthma, allergic rhinoconjunctivitis, short stature, and cerebral vascular malformations. Surprisingly, many manifestations of STAT6 GOF overlap with autosomal dominant HIES, such as eczema, high serum IgE levels, and recurrent respiratory tract infections as well as skeletal abnormalities and cerebral aneurysms. As described above, genetic defects in *STAT3*, *ZNF341*, *DOCK8*, and *IL6ST* can cause HIES, raising the suggestion of Sharma et al. to consider STAT6 GOF as another cause of HIES. The overlapping phenotype further strengthens the role of genetic testing as the most reliable diagnostic method for identifying *STAT6* variants followed by functional testing, if necessary.

### Treatment

Prior to the knowledge of the dysregulated STAT6 pathway, most patients were offered therapy with systemic corticosteroids, tacrolimus, methotrexate, and mepolizumab, which had little or no satisfactory effect on symptoms. Targeted therapies with ruxolitinib, tofacitinib, and dupilumab resulted in symptom control with improvement in atopic dermatitis, growth velocity, and eosinophilic esophagitis in numerous patients. Thereupon, total weaning from steroids was possible in some patients. Immunologically, reversion of STAT6 hyperactivation after IL-4 stimulation and faster STAT6 dephosphorylation were seen [235, 237]. Normalization of T_H_2 gene signature, IL-4Rα expression, and eosinophilia was observed.

## Homozygous *JAK1* Loss-of-Function Mutations

### Overview

For a long time, no *JAK1* LOF mutation was described, instead a perinatal lethal phenotype was shown in *JAK1* knock-out mice [242]. In in vitro experiments, JAK1 was found to play a non-redundant role, particularly in the signaling response to type I IFN (IFN-α/β) associated with TYK2 or to type II IFN (IFN-γ) associated with JAK2, underlining the distinctive role of this Janus kinase.

### Genotype

First in 2016, the only patient so far with two homozygous missense germline mutations was described, of which the P733L mutation has been undescribed and the P832S variant has already been characterized in four heterozygous individuals [243].

### Clinical Phenotype

The patient, who died at the age of 23, is characterized by various immunodeficiency symptoms (recurrent ear/chest infections, lymphadenopathy, bone abnormalities, failure to thrive, elevated IgG and IgA, leukocytosis, thrombocytosis), cardiomyopathy, pleural and mediastinal fibrosis, pulmonary macrophage infiltration, and in particular by atypical mycobacterial infections and high-grade metastatic bladder carcinoma. Susceptibility to these atypical mycobacterial infections is most likely due to T cell lymphopenia, impaired IFN-γ production by existing T cells, and a reduced JAK1-STAT1 signaling response [244].

### Molecular and Cellular Phenotype

Functional tests show reduced pSTAT levels after stimulation (low pSTAT1 after IFN-α, IFN-γ, IL-27; low pSTAT3 after IL-10; low pSTAT4 after IFN-α; low pSTAT5 after IL-2; low pSTAT6 after IL-4). T cell stimulation assays also reveal extensive immune dysregulation, especially of IFN-γ production. Further transfection experiments investigate the individual effect of the two mutations located in the pseudokinase domain, whereby P733L emerges as the probable disease-causing variant.

Eletto et al. conclude that the JAK1 pseudokinase domain, in the interaction with JAK2, takes on an essential role in the activation of the latter, allowing IFN-γ-stimulated STAT1 phosphorylation. A recent follow-up to this research addresses the question of why the patient did not suffer from viral infections (apart from flat warts) although JAK1 is significantly involved in the IFN-α-mediated immune response against viruses. It was shown that in myeloid cells JAK1 has non-redundant functions in the IFN-γ response to control intracellular bacterial infection, and - despite reduced IFN-α responses - does not lead to increased viral susceptibility in all cell types [245]. It remains to be clarified what influence JAK1 LOF has on viral susceptibility with regard to the important IFN-λ signaling pathway in epithelial cells, natural killer, and antigen-presenting cells.

## Heterozygous *JAK1* Gain-of-Function Mutations

### Overview

As early as the 1990s, mutations in the Janus kinases located upstream in the JAK/STAT signaling pathway have been described, which manifest themselves in altered signaling responses to IFN-α/β and IFN-γ [246]. Somatic activating mutations have attracted particular attention in various types of leukemia, which can be based on genetic aberrations in all four *JAK*s (*JAK1*, *JAK2*, *JAK3*, and *TYK2*) [247]. In particular, somatic GOF mutations in *JAK1* have been described in oncological pathologies such as acute lymphoblastic leukemia (ALL), acute myeloid leukemia (AML), and solid-organ cancers [248-250]. In the recent past, immunodeficiencies linked to rare germline mutations of *JAK*s have been reported, ranging from immunodeficiency to autoimmunity and lymphoproliferation. Not least because these mutations interfere so massively with the immune system, such immunodeficiencies occur very rarely and can only be identified through careful observation, detailed clinical investigation, and advanced functional analysis.

In 2017, Del Bel et al. identified the first human germline mutation of *JAK1* in a mother and her two sons, whereas previously, a somatic mutation at the identical site with a malignant phenotype was reported [251]. Phenotypically, the family was characterized by immunodysregulatory symptoms (atopic dermatitis, recurrent viral infections, autoimmune hypothyroidism, food and environmental allergies, asthma) and hypereosinophilic syndrome (eosinophilia, eosinophilic infiltration of liver and gastrointestinal tract) as well as liver cysts, hepatosplenomegaly, failure to thrive, and short stature. Since corticosteroid treatment of atopic dermatitis was not successful and no exact etiology could be identified, whole exome sequencing (WES) was conducted, and a de novo autosomal dominant heterozygous amino acid substitution mutation in *JAK1* (A634D) could be identified in the index patient and confirmed in her children. The mutation is localized in the inhibitory pseudokinase domain of JAK1, a domain which is considered to be of functional importance due to its high conservation across multiple species. Functional assays on immortalized B cells and primary CD3^+^ T cells revealed increased pSTAT1 and pSTAT3 levels both in the unstimulated setting and upon IFN and IL-6 stimulation, respectively. In vitro treatments with ruxolitinib showed normalized levels of pSTAT1 and pSTAT3, so in conjunction with the knowledge of previously described somatic mutation with a malignant phenotype [248, 252-254] and several publications of a *JAK1*^A634D^ mouse model with a similar phenotype [248, 255, 256]. Ruxolitinib was initiated as a therapy for the affected family, assuming that the mutation exerts a gain-of-function effect. This treatment was a success because it showed weight gain and reduction of eosinophils as well as resolution of dermatitis and hepatosplenomegaly.

The 2020 report of a JAK1 GOF patient with combined autoinflammatory and atopic phenotype in the case of mosaic JAK1^S703I^ genotype provided further insights into the role of the pseudokinase domain in which the mutation was localized [257]. On the one hand, the patient showed severe, early-onset, diffuse inflammation in the gastrointestinal tract, skin, and kidney, similar to the clinical pictures of STAT3 GOF or STAT1 GOF. On the other hand, the patient presented with atopy characterized by highly elevated circulating and infiltrating eosinophils, asthma, allergies, anaphylaxis, and skin disease. The mosaic phenotype due to early germline de novo mutation resulted in diffusely altered JAK1-mediated signaling, for which rather than a general hyperactivation, cell type-specific non-canonical signaling cascades with irregular expression profiles were observed with all STATs except STAT6. Large differences in the proportion of allele distribution existed between different cell types (e.g., CD14^+^ monocytes approximately 15% S703I^+^, I T cells 55% S703I^+^, CD56^hi^ NK cells 69% S703I^+^), with additional monoallelic expression bias in the transcription of JAK1 observed. The knowledge of JAK1^S703I^ mosaicism with cell type-specific allele distribution as well as the mechanism of monoallelic bias helps to understand the diffuse phenotype with different manifestation severity of autoinflammatory and atopic symptoms in different organs [258-260]. Thereby, it also provides ideas for explanations of variable penetrance in diseases such as STAT3 DN or STAT3 GOF (see above). Nearly all of the patient’s symptoms resolved under tofacitinib.

Then, in 2021 and 2022, Shomali et al. and Takeichi et al. described two more activating *JAK1* mutations. While Shomali et al. were the first to identify an acquired somatic mutation also in the pseudokinase domain (R629_S632delinsSA) with activation of the JAK1-STAT3/STAT5 pathway and clonal eosinophilic neoplasm [261], Takeichi et al. reported a heterozygous *JAK1* mutation of unclear somatic or germline origin, with both mutations showing (hyper-)eosinophilia [262]. Shomali et al. demonstrated enhanced pSTAT3 and pSTAT5 activity as well as IL-3-independent growth as a sign of Janus kinase hyperactivation in the cell line Ba/F3 used as a model for downstream signaling of kinase oncogenes. Rescue experiments with the JAK1/JAK2 inhibitor ruxolitinib however displayed normalized pSTAT5 levels and reduced growth rates. In contrast, Takeichi et al. reported the previously undescribed *JAK1* mutation H596D, which was found in a patient with a multiorgan immunodysregulatory phenotype featuring autoinflammation keratinization disease, hepatitis, autism, and atopic dermatitis. In addition, the patient presented with mild erythema, hepatosplenomegaly, failure to thrive, motor and learning impairments, glycogen storage disease type 4, and hyperlipidemia as well as lymphocytic infiltration of liver and upper dermis, epidermal hyperplasia, and eosinophilia. At the age of 3 years, acute liver failure necessitated a liver transplant; she died of unknown causes at the age of 22. A *Jak1* knock-in mouse model (*Jak1*^H595D/+;I596I/+;Y597Y/+^ mice) showed a hyperkeratotic and scaling phenotype (ears, extremities, and tail) and histopathologically lymphocytic infiltrates of the liver as well as a skin phenotype, which is overall consistent with the patient’s phenotype. By immunohistochemistry, elevated levels of pSTAT3, pSTAT6, and pJAK1 in the skin as well as pSTAT1, pSTAT3, pSTAT5, pSTAT6, and pJAK1 in the liver could be detected. Using the results of RNA sequencing of knock-in mouse samples, the authors conclude that the *JAK1* variant H596D exerts a GOF effect on the JAK/STAT signaling response [262]. Their hypothesis on pathogenesis ranges from the *JAK1* mutation and activation of inflammatory STAT-mediated signaling pathways such as the IL-6, TNF-α, or IFN-γ axis to effects such as inflammatory liver infiltration, atopic dermatitis, and eosinophilia. Since atopic dermatitis can also occur in STAT3 GOF patients (Fig. 4), the relationship of the two disease entities in terms of this complication needs to be discussed. There is little mechanistic understanding of the pathogenesis of atopic dermatitis (AD) in STAT3 GOF and JAK1 GOF. Phenotypically, AD is defined by skin inflammation, barrier dysfunction, and pruritus. In lymphocytes and dendritic cells, IL-4 can activate JAK1 via the IL-4Rα/γ_c_ complex, which leads to phosphorylation of STAT6 and STAT3. In addition to T_H_2 cell differentiation, this signaling axis is responsible for IgE synthesis of B cells and for T_H_2-targeting chemokine production (e.g., CCL17 and CCL22) by dendritic cells. STAT3 can inhibit STAT6-mediated chemokine production in these cells. In dendritic cells, IL-4 and IL-13 can activate STAT3 and STAT6 via the IL-4Rα/IL-13Rα1 receptor complex and its associated JAK1. In epidermal keratinocytes, this signaling axis leads to an increased production of T cell proliferation- and T_H_2 differentiation-promoting thymic stromal lymphopoietin, as well as IL-25 and IL-33. It also downregulates filaggrin and loricrin expression, two proteins that are indispensable to the skin barrier. The deficiency of filaggrin and loricrin, leading to the destruction of the skin barrier, is characteristic of AD. It can be overcome by the anti-IL-4Rα antibody dupilumab, which disrupts IL-13/IL-4 signaling, highlighting the central role of IL-13/IL-4 signaling dysregulation in AD pathogenesis [263]. In keratinocytes, activation of the IL-4/IL-13-JAK1-STAT3/STAT6 axis also inhibits aryl hydrocarbon receptor (AHR) signaling responses. This signaling axis acts to upregulate filaggrin and other barrier-related proteins, ensuring a protective skin barrier. The inhibition of IL-4/IL-13-mediated STAT6 phosphorylation in turn leads to decreased filaggrin expression by STAT3 inhibition via periostin and IL-24. The degree of barrier function is thus determined by a balanced expression of barrier proteins, which is a result of the upregulating AHR axis and the downregulating IL-13/IL-4-STAT3/STAT6 axis. A reduction in IL-13 levels is accompanied by an improvement in skin permeability, a reduction in T_H_2 chemokines, and a restoration of barrier-related protein expression. Therefore, in addition to corticosteroids, the IL-4Rα antibody dupilumab and JAK inhibitors have been tested for treatment. The topical JAK inhibitor delgocitinib is approved in some countries for the treatment of atopic dermatitis. By interfering with the IL-4Rα/γ_c_, IL-4Rα/IL-13Rα1, and IL-31RA/OSMR signaling responses, skin lesions, associated itch, and the skin barrier itself can be reliably controlled. In the future, instead of steroids, combined treatment of IL-13/IL-4-JAK-STAT6/STAT3 axis inhibitors together with IL-13/IL-4 interfering antibodies may be used. As STAT3 has been shown to take on similar functions as STAT6 in lymphocytes and keratinocytes, one might learn from the STAT6 GOF genotype. Overexpression of STAT6 in mice is also associated with AD-like skin symptoms, which is consistent with atopic symptoms in STAT6 GOF patients. Based on this knowledge, AD can also be understood as cutaneous inflammation due to elevated IL-13/IL-4 signaling, which might be related to dysregulated JAK1 and STAT3 responses.

It is interesting to note that the four variants described were all found in the pseudokinase domain of JAK1. In addition, somatic *JAK1* mutations leading to a malignant phenotype such as T cell neoplasms, ALL, myeloproliferative neoplasm [264-266], AML, or other solid-organ cancers [248-250] have been described. For example, in T-ALL, activating mutations in *JAK1* are described to be found in association with mutations in other genes, such as *NOTCH1* or *PTPRC* [248, 267]. Similarly, *JAK3* GOF mutations can be found concordantly with mutations in *TP53*, *NOTCH1*, or *CDKN2A* [268]. Such concurrent mutations are thought to act synergistically to promote the proliferation or survival of T cell leukemic cells, as well as to disrupt normal T cell differentiation. The co-occurrence of *JAK1* and *JAK3* mutations with other genetic alterations has also been observed in solid cancers, such as breast cancer. In melanoma, mutations in *BRAF* have been found to accompany *JAK3* mutations [269], while in breast cancer, mutations in such as *TP53* and *NOTCH1*, as well as *MYC* amplification, have been identified alongside *JAK1* mutations [270]. This cooperation of additional mutations is thought to play a mechanistic role in the development and progression of cancer by facilitating cellular proliferation, invasion, and metastasis, suggesting that the presence of *JAK* mutations in addition to other known oncogenes is likely promoting tumorigenesis. In addition, mutations of the pseudokinase domain of JAK2, a structural homologue of JAK1, are found in patients with chronic eosinophilic leukemia and a consequent polycythemia [271]. It can be assumed that a tightly regulated signaling response by JAK1 and JAK2 is essential for immune homeostasis and especially for eosinophil survival. Trials on the JAK1/JAK2 inhibitor in primary eosinophilic neoplasms and idiopathic hypereosinophilic syndrome are currently in clinical phase 2. In particular, the long-term effects of ruxolitinib therapy on the human organism have not yet been sufficiently investigated; conceivable side effects may include myelosuppressive anemia. For this purpose, the development of highly selective JAK inhibitors, such as the latest generation of jakinibs, would be optimal [272, 273]. However, selectivity in JAK inhibitors is a relative term due to the high structural homology of the JH1 domain of JAK isoforms, which is the active catalytic domain targeted by JAK inhibitors. Different JAK inhibitor molecules show different association rates to JAK1, JAK2, JAK3, and TYK2, which is measured in vitro as inhibitory concentration IC50; therefore, never 100% specificity is achieved. Furthermore, the clinical effect of JAK inhibitors is dose and cell-type dependent, the first itself being a modulator of isoform specificity [274]. In particular, the cell type-specific receptor profile, various coupling patterns of JAK heterodimers, and hierarchical activation between JAKs in different receptors lead to an unfathomable complexity in the signaling pathways downstream of the Janus kinases [275]. This results in different selectivities of JAK inhibitors in different cytokine pathways. The fact that the inhibition of a JAK directly suppresses numerous cytokine signaling pathways, which in turn influence the production of JAK-independent signaling pathways such as the IL-17/Act axis, underlines the broad indirect spectrum of action of even highly selective JAK inhibitors. Nevertheless, selective jakinibs such as the JAK1 inhibitors filgotinib, upadacitinib, itacitinib, and solcitinib are considered to cause less interference to off-target cellular functions and therewith reduced adverse effects. Even with highly selective JAK inhibitors, however, we will still have to deal with an adverse effect spectrum similar to that of pan-JAK inhibitors. This is also due to the multiple interactions of JAK1 with all other Janus kinases (JAK1/JAK2 via IFN-γ receptor and glycoprotein (gp) 130-containing receptors, JAK1/JAK3 via γ_c_ receptor, JAK1/TYK2 via IFN-α/β receptor and gp130 receptors).

## Homozygous and Compound Heterozygous *JAK3* Loss-of-Function Mutations

### Genotype

A few years after the discovery of mutations in the *IL2RG* gene encoding the common gamma-chain (γ_c_) as the cause of X-linked severe combined immunodeficiency (X-SCID), an immunodeficiency characterized by the absence of T cells and NK cells in the presence of non-functional B cells [276], *JAK3* mutations were identified as the cause of autosomal recessive T^−^B^+^NK^–^SCID [277, 278]. Between 7 and 20% of all congenital SCID cases are caused by predominantly compound heterozygous *JAK3* mutations [279-281]. In addition, the JAK3/STAT signaling pathway can be impaired by mutations in JAK3-associated receptors such as *IL7R* (IL-7Rα) (< 10%) or *IL2RG* (γ_c_) (25–46%) [282].

### Molecular and Cellular Phenotype

Pathomolecularly, impaired T and NK cell development and B cell function may be due to reduced or abolished protein expression, protein stability, or receptor binding of the JAK3 protein, which physiologically interacts with γ_c_ and IL-7Rα to mediate the signaling response of interleukins IL-2, IL-4, IL-7, IL-9, IL-15, and IL-21 acting through this pathway [283]. Contrary to mice, not the complete lymphocyte development is impaired, but only the T cell and the NK cell development since these cell types depend on IL-7Rα/γ_c/_JAK3 signaling [284].

### Clinical Phenotype

As a result of the defect, patients who appear healthy for a short time after birth suffer from a massive restriction of the immune defense; therefore, this clinical picture must be considered life-threatening. Humoral and cellular immune responses are extremely reduced due to the absence of T cells, resulting in a life-threatening infection susceptibility. In particular, recurrent or persistent infections of the respiratory tract and gastrointestinal tract with viruses and fungi (initially less frequently bacteria because of the maternal passive immunity in infancy), chronic infections with opportunistic pathogens, chronic diarrhea, and failure to thrive determine the fatal outcome of the disease in early childhood if not treated adequately [285].

### Treatment

To protect patients from ubiquitous pathogens, infants used to be isolated in sterile plastic tents, so they were known as “bubble babies”. Fortunately, over the last decades, HSCT has been optimized as the curative therapy of choice to such an extent that a long-term survival of up to 95% can be achieved with an optimally matched donor-recipient pairing. Compared to other forms of SCID with B cell loss, the presence of B cells has a positive effect on the outcome of HSCT [286]. Since HSCT may be accompanied by side effects of immunosuppression and GvHD, gene therapy approaches with retroviral vectors were developed for X-SCID patients. So far, such treatment options for JAK3 deficiencies leading to SCID have not been described; however, due to the pathomolecular and clinical similarities between X-SCID and JAK3-SCID, it seems promising to genetically correct autologous stem cells of affected individuals using new molecular tools such as CRISPR/Cas9 and thus establish a new curative treatment option.

## Heterozygous *JAK3* Gain-of-Function Mutations

Activating germline mutations of JAK3 were previously undescribed, only somatic GOF mutations were known to exist in T cell proliferative leukemia [287, 288], though most recently three patients with inborn *JAK3* GOF mutations have been reported. The novel germline heterozygous *JAK3* variant Q507P in a mother and her son was phenotypically associated with vasculitis and CVID, recurrent multilineage autoimmune cytopenia, psoriasis, hypogammaglobulinemia, and NK cell lymphoproliferation as well as elevated numbers of aberrant NK cells, large granular lymphocytes with benign morphology, reduced memory, and isotype-switched B cells [289]. The Q507P variant in the linker domain is known to be a somatic mutation causing T cell prolymphocytic leukemia [290] and was accordingly classified as a GOF variant. Functional studies revealed constitutive phosphorylation in an in vitro cell model and increased pSTAT5 levels in NK cells after IL-2 stimulation of primary lymphocytes, which was therefore defined as a mechanism of benign NK cell lymphoproliferation leading to PID.

In a second case report, the rare heterozygous *JAK3*^R840C^ variant was first found in association with a new heterozygous LOF *CTLA4*^Y139C^ variant with the clinical picture of PID. As the mutation localizes within the JH1 domain in close proximity to the ATP-binding site, Sic et al. suggest a possible altered N-lobe conformation and interaction with the pseudokinase domain resulting in overactivation of the protein [291]. When combined with the CTLA4 LOF effect, the variant - relatively mild compared to other activating mutations such as *JAK3*^A572V^ - stands out and manifests in an immunodysregulatory phenotype. The latter is characterized by early bronchiolitis, recurrent upper respiratory tract infections, lymphadenopathy, splenomegaly, pancytopenia, severe hypogammaglobulinemia, insufficient vaccination response, hypothyroidism, and hypercellular bone marrow. The authors show that a large proportion of the present T cells are CXCR5^+^PD-1^+^TIGIT^+^ T_FH_ cells, which, however, can only insufficiently produce the cytokines necessary for plasma cell and memory B cell maturation. The combination of the individually harmless *JAK3* and *CTLA4* variants results in a T_FH_ cell defect with symptoms such as lymphadenopathy, autoimmunity, and hypogammaglobulinemia.

## Clinical Relevance and Consequences for Therapeutic Approaches

The integration of clinical studies and basic science research creates an understanding of the pathomolecular processes and thus allows patient-centered and individualized therapies. In terms of autoimmune diseases, JAK inhibitors have already successfully been introduced into clinic (e.g., tofacitinib for rheumatoid arthritis, baricitinib for atopic dermatitis) [292]. These inhibitors suppress the upstream JAK molecules in order to prevent an excess of an activated STAT signal, making them causal therapeutics for hyperactivated JAK/STAT activity, e.g., in case of STAT GOF mutations but potentially also in case of SOCS dysregulation. As with many drugs that intervene in such a pivotal control mechanism of the cell, it is particularly crucial to find the equilibrium between efficacy and side effects. Fortunately, the short half-life of jakinibs (8–12 h) allows for an easy titration of the dose. However, recent studies reveal the relevant side effects of JAK inhibitors, highlighting the need for individualized benefit-risk evaluation before JAK inhibitor therapy [293, 294]. Differences in reporting of side effects between JAK inhibitors were observed, for example, while tofacitinib (JAK1/JAK3 inhibitor) generates cardiovascular and cancer effects as well as gastrointestinal perforations, baricitinib (JAK1/JAK2 inhibitor) caused more embolism and thrombosis, and ruxolitinib (JAK1/JAK2 inhibitor) caused more malignant hematopoietic and skin neoplasia. To varying degrees, the three jakinibs caused viral (herpes or influenza), fungal (*Pneumocystis*, *Cryptococcus*, or *Coccidioides*), and mycobacterial infections (tuberculous and atypical). Thus, rational use of JAK inhibitors must be considered when treating STAT1 GOF or STAT3 GOF patients, in which the risk for viral and fungal infections or cancer is already elevated, respectively, in those patients.

In addition to the small molecules inhibiting JAK directly, there are also interleukin inhibitors and interleukin-receptor inhibitors (mostly antibodies), STAT inhibitors, and SOCS mimetics, which are currently at different stages of development (preclinical and clinical studies). Oral STAT3 inhibitors have already been developed for solid or hematopoietic tumors but have not yet entered clinical use due to severe adverse effects and poor selectivity. As many mutations are located in the SH_2_ domain, one research focus lies on the development of non-peptide or peptidomimetic agents to disturb SH_2_ domain interactions with other STAT molecules as well as inhibition of tyrosine and serine phosphorylation. Further obstacles are poor bioavailability of peptide agents and side effects in the form of, for example, cytopenia and dose-limiting vomiting with the STAT3 inhibitor OPB-51602 and OPB-31121. In addition to N-terminal inhibitors, DNA binding domain inhibitors and decoy oligodeoxynucleotides are also being investigated; the latter are designed to mimic the STAT DNA binding sequence in order to sequester STATs and downregulate STAT-dependent gene transcription. Similar approaches are being pursued with small interfering RNAs and antisense oligonucleotides to induce STAT degradation. Since this region is highly conserved between STATs, unselectivity such as off-target STAT1 blockade by a STAT3 inhibitor remains a challenge in development [295-298].

In the last decade, in vitro research has been performed to develop SOCS mimetics and SOCS antagonists, which have shown encouraging results in mouse models of autoimmune-associated diseases like diabetes and multiple sclerosis. The underlying design principle involves the development of a peptide that contains the kinase inhibitory region (KIR), which is naturally present in SOCS1 and SOCS3. Physiologically, KIRs take over the main role of SOCS1 and SOCS3 in inhibiting JAK2, so that the activation of downstream targets such as STAT1 is reduced. In this way, the use of small peptide SOCS mimetics containing KIR sequences allows the immune balance to be shifted toward inhibition of cytokine responses with the consequence of reduced autoimmunity. Conversely, SOCS antagonists, which act mainly via proteolytic degradation, can promote the expression of proinflammatory cytokines via enhanced STAT signaling responses, thereby enhancing viral immunity through, e.g., SOCS1 inhibition. So far, there are no known clinical trials for SOCS mimetics or antagonists. Similar problems in development as with JAK inhibitors and STAT inhibitors are anticipated: The KIR sequences of SOCS1 and SOCS3 are partially homologous, so cross-regulation is feasible. An essential part of the development of such therapeutics will be the balance of homeostasis between excessive and insufficient immunity in an already dysregulated organism via phenotype-adapted dose-specific SOCS regulation [299-301].

Up to know, there are no therapies available for an attenuated JAK/STAT signal (e.g., due to a *STAT3* DN mutation) apart from stem cell transplantation, which carries considerable side effects and risks. The immunosuppressive effects of conditioning therapy used in HSCT increase the infection susceptibility of patients, which can cause significant morbidity. Prophylactic measures such as antibiotics and immunoglobulin therapy are necessary to prevent these life-threatening infections. While with GvHD, the graft immune system attacks host tissue, failure to engraft is another complication, leaving the patient immunocompromised and in need of a second HSCT. The side effects of conditioning therapy with chemotherapeutic agents to deplete bone medullary stem cells, among others, can be diverse and serious, possibly with long-term consequences for vulnerable organs such as the brain, lungs, kidney, and liver. The comparatively rare use of HSCT in germline JAK/STAT diseases with unknown long-term efficacy must be taken into account. Obviously, non-hematological disease manifestations (such as skeletal abnormalities in STAT3 DN) or irreversible remodeling processes (such as bronchiectasis/pneumatoceles in STAT3 DN or vascular changes in STAT1 GOF) cannot be successfully treated by HSCT. Consequently, in order to consider HSCT for diseases with high disease-related morbidity and mortality (such as STAT1 LOF), an early disease diagnosis including prognosis assessment is indispensable. Across the different JAK/STAT diseases, different outcomes are reported with about 30% survival in STAT3 GOF, 40% in STAT1 GOF, 70% in STAT1 LOF to up to 95% in JAK3 LOF. In general, the risk of disease progression and disease complications associated with HSCT and mortality must be weighed individually for each patient, and a decision must be made for the most beneficial therapy [302, 303]. Currently, bridging therapies such as ruxolitinib in STAT1 GOF are being studied to minimize the risk of secondary graft failure and thereby improve survival [230].

The final goal will most likely be the replacement of stem cell transplantation by gene therapeutic approaches. To enable this, the disease-causing LOF mutations could be replaced by DNA sequences that encode for the healthy sequence of the affected proteins. For GOF mutations, however, theoretically gene therapy needs to be preceded by a full conditioning of the recipient’s bone marrow to allow for only corrected cells to repopulate the immune system. Personalized medicine would then become established in the field of the JAK/STAT signaling pathway, which would help many patients to improve their quality of life.

## Shifting our Understanding from a Continuum to a Two-Dimensional Concept

Since the early beginning of immune research in the mid-twentieth century, immunodeficiency and autoimmunity are terms used to describe impaired infection resistance and disturbed activation of the innate or acquired immune system [304]. The terms help clinicians to assign syndromes to categories and thus enable specialized therapy. For instance, in immunodeficiencies such as CVID, and also HIES, immunoglobulins are used for the treatment and prevention of respiratory infections due to a lack of antibodies. Until today, the JAK/STAT diseases discussed above have been classified on a spectrum between the two extremes of immunodeficiency vs. autoimmunity/autoinflammation. Recent scientific reports are beginning to recognize that the former concept of loss-of-function mutations are always associated with immunodeficiency and gain-of-function mutations are only associated with autoimmunity and autoinflammation is no longer clinically tenable (Fig. 4) [305, 306]. This simplistic understanding is outdated and instead a new way of thinking about STAT1, STAT3, STAT6, JAK1, and JAK3 LOF and GOF disease in terms of the relationship between immunodeficiency and autoimmunity is developing (Fig. 2). Reviewers are starting to argue that autoimmunity and immunodeficiency are “two sides of the same coin”. Recent patient case reports and an improved (patho-)molecular understanding of pathway dysregulations help to explain the sometimes clinically contradictory symptoms (Fig. 4).

We want to outline the seemingly paradoxical phenotypes once again in order to gain an understanding of how our perception of JAK/STAT diseases has changed:

First, in 2021, the lupus-like autoimmune phenotype was described in a cohort of ten STAT3 DN patients [62]. A new symptom cluster has emerged, which was previously perceived exclusively with an autoimmune phenotype. This specific phenotype clinically manifests with nephritis, autoimmune cytopenia (ITP, AIHA, leukopenia), discoid rash, and alopecia. Notably, all ten patients had antinuclear antibodies, with 9 and 7 patients having anti-double-stranded DNA autoantibodies and reduced C3 and/or C4, respectively. Remarkable across all STAT3 DN patients was the increased expression of ISGs (including *HERC5*, *IFI44L*, *MX1*, *RSAD2*, *IFI27*, and *CXCL10*), NET formation, and anti-NET autoantibodies. NETs are extracellular formations of mainly neutrophil DNA that bind pathogens and are therefore considered one of the major defense mechanisms of neutrophils. Nowadays, NETs are considered to have a significant role in the etiology of lupus autoantigens and in driving proinflammatory mechanisms, thus providing a pathomechanistic link between autoimmunity and STAT3 DN in this context [307]. Moreover, NETs are now considered to be highly interferogenic, which may explain the increased IFN production in STAT3 DN patients [62]. It remains to be clarified why almost all STAT3 DN patients show such molecular mechanisms, but only 6.8% present with an autoimmune phenotype.

Second, the main characteristics of STAT3 GOF are autoimmune cytopenia, lymphadenopathy, hepatosplenomegaly, autoimmune enteropathy, interstitial lung disease, autoimmune hypothyroidism, type I diabetes mellitus, and other inflammatory manifestations such as AD. Nevertheless, it is now known that cellular and humoral immunodeficiencies occur in 37% and 51% of patients, respectively [308]. Advanced models exist to elucidate autoimmune processes via dysregulation of T_regs_ in STAT3 GOF. The pathogenesis of increased susceptibility to infections caused by bacteria, viruses, mycobacteria and fungi is largely not understood. Although decreased counts of dendritic, T_reg_, T_H_17, and NK cells are measured in patients, molecular mechanisms behind the increased infection susceptibility in the presence of, e.g., normal NK cell function (with normal IFN-γ receptors and normal IFN-γ production), are not understood. The mechanisms of deficiency of switched memory B cells resulting in hypogammaglobulinemia are also not yet understood [97]. Interestingly, the STAT3 signaling pathway was observed to interfere with STAT1 function [88]. This is based on similar misdirected regulatory mechanisms as described before, such that increased STAT3 function induces increased SOCS3 activity and, via cross-regulation, impairs STAT1 function as well. Ultimately, this is thought to mimic a STAT1 LOF phenotype known to be associated with recurrent mycobacterial and viral infections.

Third, almost every STAT1 GOF patient will develop CMC in his or her lifetime, predominantly as a result of *Candida albicans* infection [208]. Not until the discovery of the STAT1 genotype in the last decade was it clear that, conversely, about half of all CMC cases are attributable to heterozygous STAT1 mutations [164, 165]. The pathomechanisms behind this immunodeficiency are still being investigated, but it is known that T_H_17 cells play an important role in the immunity against *C. albicans*. Therefore, in the case of STAT1 hyperactivation, there is enhanced IL-27-mediated upregulation of PD-L1, which inhibits T_H_17 differentiation. Altered STAT3 promoter binding through STAT1 GOF-dependent epigenetic alteration of acetylation status also plays a role in IL-17 dysregulation by decreasing STAT3-dependent transcription that facilitates T_H_17 differentiation (RORγt/IL-17/IL-22/IL-10/c-Fos/SOCS3/c-Myc) [208]. Notably, despite reduced IL-17 immunity, which is typically known as a potent inducer of tissue inflammation and autoimmunity, STAT1 GOF may also present with symptoms such as autoimmune thyroid disease, autoimmune cytopenia, psoriasis, and other autoimmune symptoms referred to as the IPEX-like phenotype. Molecular explanations remain at a conceptual level. It is assumed that the T_regs_ necessary for the control of the immune responses play a relevant role in the IPEX-like syndrome, but functional tests and T_reg_ counting have not yet provided any significant evidence for this hypothesis. The clinical picture of STAT1 GOF exemplifies how the seemingly paradoxical coexistence of immunodeficiency and autoimmunity is reality for many patients. Until now, the mechanisms are still poorly understood. We are left with the question on the different penetrance of the different symptom complexes: why do almost all patients develop CMC and bacterial infections, but less than half develop autoimmune/autoinflammatory symptoms?

This new understanding implies three important aspects that will influence the management of JAK/STAT diseases in the future: There is a need to acknowledge that certain mutations in proteins offer great possibility for dysregulation not only of the canonical pathway, but also of many pathways that are as yet poorly understood or not recognized at all (Fig. 3). Through epigenetic changes, far-reaching dysregulations are also possible in intricate fields such as transcriptional profiles. The knowledge of emerging pathological mechanisms through which phenotypes can manifest themselves must sensitize clinicians with regard to symptom diversity. Pathways with partly antagonistic functions can be affected in individual cases and thus lead to an ambiguous clinical picture that can significantly complicate a precise diagnosis. In the context of rare diseases, up-to-date knowledge of all phenotypes, whether immunodeficiency, autoimmunity, or both, is essential to enable effective differential diagnosis (e.g., as in SLE-like phenotype in STAT3 DN).

Further, this creates a need for meticulous case reports when novel mutations are identified and reviews to identify clustered symptoms that may be rare. Moreover, this requires derived functional studies that investigate the molecular pathomechanisms behind the clinical phenotype (e.g., as in IPEX-like phenotype in inconspicuous T_regs_). Only through a deeper understanding of the pathomechanism at the molecular level it will be possible to develop effective causal therapies (beyond non-specific immunosuppression or immunoglobulin administration). JAK inhibitors have ushered in a new era of immunotherapies, with the ability to be truly targeted in the case of JAK/STAT deficiencies. Integral personalized therapy is only possible if both immunodeficiency and autoimmunity are addressed in treatment when they co-occur. In such cases, dose titration makes sense, as does the use of highly specific JAK inhibitors as opposed to broad-acting ones to treat only the dysregulated protein or pathway. In the future, there will probably also be - so far paradoxical - therapeutic approaches, such as JAK inhibition in SLE-like phenotype in STAT3 DN [63]. One can only imagine the possibilities that could be opened up by therapeutics such as SOCS mimetics or STAT inhibitors if defects were treated as proximal as possible in the signaling pathway. Any further downstream misregulation with immunodeficient and autoimmune manifestations could be averted in this way. Thinking big, advanced causal approaches such as gene therapy will provide future opportunities to get to the root of a genetic defect, therefore enabling true restoration of molecular processes.

## Conclusion

Since the JAK/STAT signaling pathway plays a central role in mediating fundamental biological processes such as in development and in the immune system, genetic defects in these players may lead to dramatic clinical consequences. Over the past years, new results in JAK/STAT research improved the pathomolecular understanding of the syndromes. Case reports of new patients with, e.g., JAK1 GOF, reveal novel disease patterns that were previously undetected due to their rarity or their life-threatening nature. The improved pathophysiological understanding leads to focused diagnostic procedures and targeted treatment approaches as seen in STAT6 GOF. The reporting of more patients with rare diseases is essential to systematically identify sporadic complications (such as mycobacterial and viral infections in autosomal recessive complete *STAT1* LOF mutations) and to apply them in the diagnosis of new patients. A far-sighted and open-minded research attitude is of great benefit if unexpected results (such as SLE-like autoimmunity in *STAT3* DN mutations) can be assigned to these diseases and new explanatory approaches for a holistic understanding of the syndromes arise. For several years now, JAK inhibitors have also been able to be used selectively in cases of overactivation of the signaling pathways, and more causal approaches such as HSCT and, in the future, gene therapies are developing. For all therapies, the risk of adverse events must be considered, such as increased incidence of viral infections in STAT1 GOF during JAK inhibitor use or fatal outcomes in HSCT of STAT3 GOF patients. This review provided a distinguished overview of recent findings of syndromes associated with *STAT1*, *STAT3*, *STAT6*, *JAK1*, or *JAK3* germline mutations emphasizing the relation of clinical signs, symptoms, laboratory findings, and the pathomolecular etiology. The understanding of JAK/STAT dysregulation as a model on a one-dimensional spectrum between the extremes of immunodeficiency and autoimmunity is now becoming outdated. Instead, progress in pathomechanistic understanding and clinical and immunological manifestations is leading to multidimensional representations that reconcile the coexistence of symptoms from both ends of the immunity spectrum. We therefore advocate a two-dimensional understanding of JAK/STAT LOF and GOF diseases, with the intention of identifying novel molecular pathomechanisms, to discover and provide explanations for previously opaque phenotypes and to develop new therapeutic approaches for affected patients.

## Data Availability

Not applicable.
